# Electrical Remodeling of Pressure Overloaded Rat Heart Is Attenuated if Imposed During Proliferative Cardiac Growth

**DOI:** 10.1111/apha.70118

**Published:** 2025-10-15

**Authors:** Eva Nekvindova, Jaroslav Hrdlicka, Almos Boros, Michaela Slegrova, Alena Kvasilova, Vojtech Skop, Jan Halberstat, Kristyna Holzerova, Jan Neckar, David Sedmera, Veronika Olejnickova

**Affiliations:** ^1^ First Faculty of Medicine Institute of Anatomy, Charles University Prague Czech Republic; ^2^ Institute of Physiology, Czech Academy of Sciences Prague Czech Republic; ^3^ Centre for Experimental Medicine, Institute for Clinical and Experimental Medicine Prague Czech Republic; ^4^ Department of Biochemistry and Microbiology University of Chemistry and Technology Prague Czech Republic; ^5^ Czech Centre for Phenogenomics, Institute of Molecular Genetics of the Czech Academy of Sciences Vestec Czech Republic

**Keywords:** arrhythmias, connexin 43, electrophysiology, hyperplasia, hypertrophy, left ventricle pressure overload, neonatal rat

## Abstract

**Aim:**

Left ventricular pressure overload (LVPO) in adults is associated with adverse electrical remodeling, characterized by reduced conduction velocity (CV). However, the progression of LVPO differs when imposed during the proliferative phase of cardiac development. It remains unknown how increased cardiomyocyte proliferation affects LVPO electrical remodeling.

**Methods:**

CV maturation from rat postnatal day (PD) 1 to PD90 and analyzed underlying connexin 43 (Cx43) profile. Pressure overload was induced by abdominal aortic constriction (AAC) in rats during the proliferative phase of cardiac growth (PD2). Animals subjected to AAC during the non‐proliferative heart growth (AAC‐PD6) and Sham‐operated rats served as controls. Electrical remodeling was assessed at PD21 using ECG, optical mapping, western blots, immunofluorescence, and lipidomic analysis, complemented by functional analyses through echocardiography.

**Results:**

Pressure overload led to a 2.5‐fold increase in heart weight compared to Sham in both AAC groups. A significant increase in relative left ventricular wall thickening was observed in AAC‐PD2 rats only. Optical mapping and ECG showed preserved conduction properties in AAC‐PD2 animals, whereas the AAC‐PD6 group displayed prolonged QRS and significantly reduced longitudinal CV. While total and phosphorylated Cx43 levels were comparable between the AAC groups, AAC‐PD2 animals demonstrated higher intercalated disc localization. Furthermore, lipidomic profiling revealed maintained long‐chain acylcarnitine (LCAC) levels in AAC‐PD2, whereas AAC‐PD6 tended toward LCAC accumulation.

**Conclusion:**

This study provides new insights into the remodeling upon pressure overload during cardiac proliferative growth, demonstrating attenuated electrical alteration by preserved CV and highlighting the role of Cx43 localization and preserved levels of LCACs.

## 1 Introduction

Left ventricular pressure overload (LVPO) primarily corresponding to the elevated blood pressure and manifested as left ventricular hypertrophy (LVH) represents a global health challenge with important adverse outcomes [1, 2]. In adults, LVPO leads to heart enlargement to compensate for increased afterload [3, 4]. Since there is a limited possibility for cardiomyocyte proliferation in adulthood [5, 6], cardiac growth is realized through hypertrophy of individual cardiomyocytes. Although LVH enables the adult heart to adapt to elevated pressure load, it is also associated with adverse electrical remodeling characterized by dysregulation of gap junction channels and alterations in ion channel function [7, 8, 9, 10]. As a result, impulse conduction slows, leading the myocardium to be more susceptible to potentially life‐threatening arrhythmias and sudden cardiac death (SCD) [8, 9, 11]. The Framingham study reported that each increment in left ventricular (LV) mass correlates with a 1.5‐fold increase in cardiovascular disease risk and a 1.7‐ to 2.1‐fold increase in risk‐adjusted cardiovascular mortality [12, 13, 14, 15, 16].

Under physiological conditions, electrical impulses propagate through the ventricular myocardium via intercellular coupling mediated by connexin 43 (Cx43) gap junction channels. Any disruption of these low‐resistance communications, namely Cx43 downregulation, functional dephosphorylation, heterogeneous expression, or lateralization (relocation from their dominant position at the intercalated disk (ICDs) to the lateral membrane of cardiomyocytes), is associated with reduced conduction velocity (CV) and increased susceptibility to ventricular arrhythmias [17, 18, 19]. These alteration at the Cx43 level seems to be general mechanisms since it was described in cardiac diseases of various etiology [20, 21]. The deterioration in electrical conduction is ultimately exacerbated by increased collagen deposition [22, 23] and alteration of the cardiac lipid spectrum [24, 25].

In contrast to adults, neonatal hearts in the early postnatal period possess unique features that influence their response to pathological stimuli [26, 27]. Retained proliferative capacity enables myocardial regeneration after injury [28, 29]. Rodent models of apical resection and neonatal myocardial infarction have shown complete regeneration of injured myocardium when injury is imposed during the first postnatal days [30, 31, 32]. Given that myocardial conduction depends on effective intercellular coupling, it remains unclear how increased cardiomyocyte number induced by neonatal LVPO affects electrical remodeling. This question is particularly compelling, as neonatal cardiomyocytes exhibit a distinct Cx43 distribution. While Cx43 is predominantly located at the intercalated discs in adult hearts, it is distributed more uniformly along the cell membrane in neonatal cardiomyocytes [33, 34]. However, the timing and pattern of Cx43 relocation during postnatal development remain poorly understood. Since congenital heart diseases (CHDs) associated with elevated afterload account for a significant proportion of CHDs [35, 36], understanding how electrical remodeling is modified when it occurs during the proliferative phase of cardiac growth is clinically important.

A model of abdominal aortic constriction (AAC) in 2‐day‐old rats [37, 38, 39] (AAC‐PD2), which has been documented to induce cardiomyocyte proliferation, was employed to better understand the mechanisms of electrical remodeling during the proliferative phase of growth [29, 39]. As an initial step, we characterized the relationship between conduction properties and Cx43 redistribution during postnatal CV maturation.

## 2 Results

### 2.1 Maturation of the Conduction Parameters and Related Connexin 43 Localization

**FIGURE 1:**
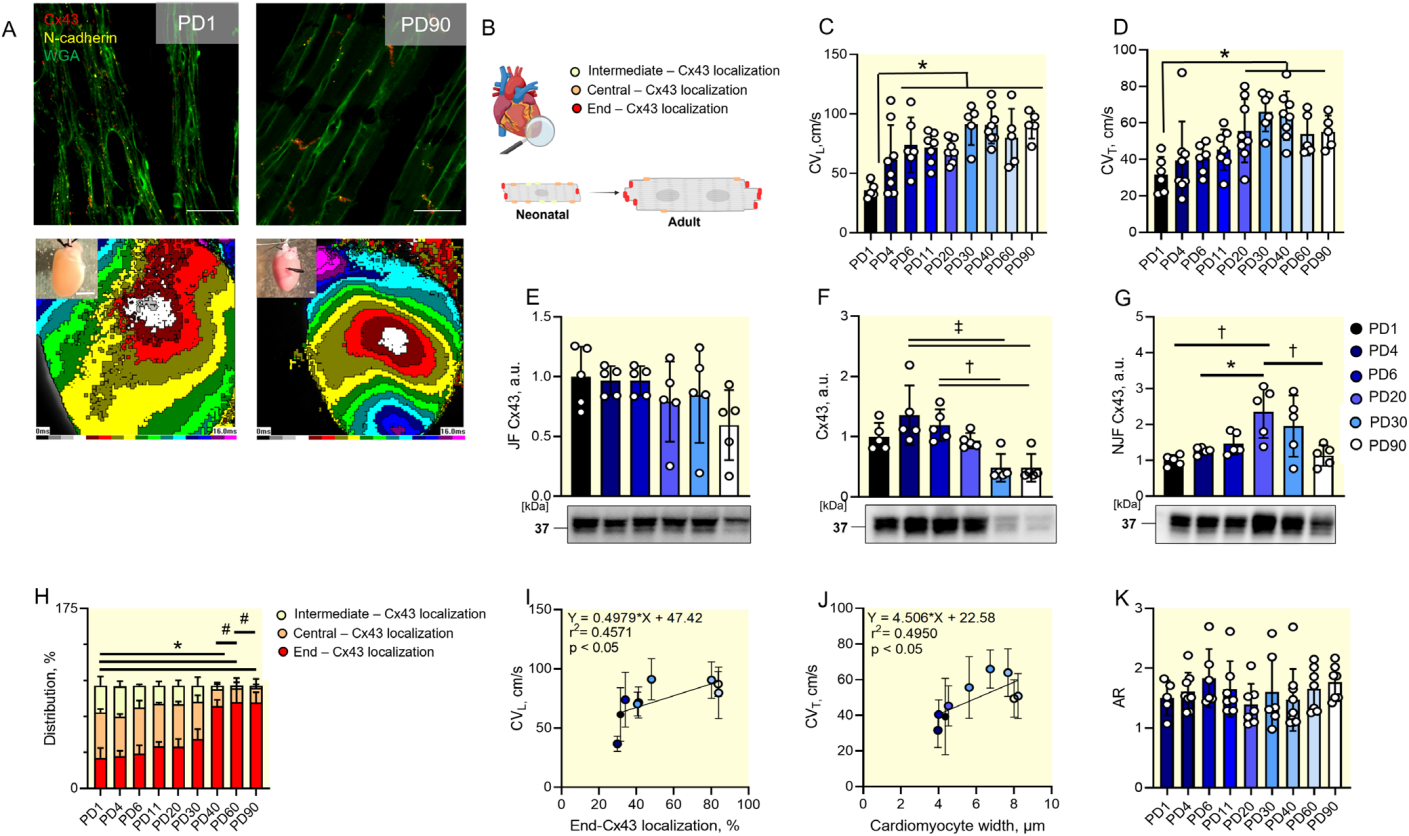
Maturation of the conduction parameters and related Cx43 changes during postnatal development. Representative images of the circular layer of the left ventricular free wall showing localization of Cx43 (red) in relation to the marker of intercalated discs—N‐cadherin (yellow) (wheat germ agglutinin staining of the myocyte membrane in green) with corresponding optical maps and heart size at the postnatal day (PD) 1 and PD90 (A). Schematic representation of Cx43 distribution at the cardiomyocyte border at neonatal and adult stages (B). Quantification of the longitudinal (C) and transversal conduction velocity from optical mapping; *n* = 5–9 animals per group (D). Cx43 protein expression normalized to total protein in the junctional fraction (E), total amount of the Cx43 (F) and in non‐junctional Cx43 fraction (G) across development stages; *n* = 5 animals per group. Quantification of the Cx43 distribution at specific parts of the cell border detected by immunohistochemistry during development; *n* = 5 animals per group (H). Correlation between end‐localized Cx43 and longitudinal conduction velocity; *n* = 5 animals per group (I) and between cardiomyocyte width and transversal conduction velocity; *n* = 5 animals per group (J). Quantification of the anisotropy ratio across the examined developmental stages, *n* = 5–9 animals per group (K). AR, anisotropy ratio; CV_L_, longitudinal conduction velocity; CV_T_, transversal conduction velocity; JF, junctional fraction; NJF, non‐ junctional fraction; PD, postnatal day. Scale bar 25 μm for microscopy images and 2 mm for macroscopy images. Data are expressed as mean ± SD; One‐way ANOVA with Tukey multiple comparisons test was used for C–G and K, *p < 0.05, †*p* < 0.01, #*p* < 0.001. Chi‐square test was used for H, *p < 0.05 vs. PD1, †*p* < 0.05 vs. previous developmental stage. Pearson correlation coefficient was used for I, J.

Although postnatal cardiac ultrastructural remodeling is well‐documented [27, 40], changes in conduction properties during this period remain insufficiently characterized. Therefore, optical mapping of the paced Langendorf perfused isolated heart was employed to analyze longitudinal and transversal conduction velocity (CV_L_, CV_T_; Figure S1) from PD1 to PD90 (Figure 1A,B). Both CV_L_ and CV_T_ increased with age; however, they exhibited distinct developmental dynamics. CV_L_ showed a stepwise increase, with a significant elevation between PD1 and PD4 (*p* < 0.05). This increase was followed by stable values until PD30, at which point adult levels were reached and remained unchanged through PD90. At PD90, CV_L_ was 238% of its PD1 value (Figure 1C). In contrast, CV_T_ increased more gradually and reached significance relative to PD1 at PD20. The total increase in CV_T_ at PD90 was 158% of the PD1 value (Figure 1D).

To determine the structural basis for these changes, we analyzed total Cx43 and its subcellular distribution; that is, membrane‐bound (junctional fraction, JF) and cytoplasmic (non‐junctional fraction, NJF). No significant differences were observed in the JF across development (Figure 1E), suggesting that membrane‐associated Cx43 levels exhibited minor fluctuations throughout postnatal maturation. However, total Cx43 and NJF exhibited marked changes throughout development. The total Cx43 amount was significantly elevated at the PD4 and PD6 from PD30 and 90 (*p* < 0.01 and 0.001; Figure 1F). The highest expression of Cx43 in NJF was reached at the PD20 and was significantly increased from the PD1, PD4, and PD90 (*p* < 0.05 and 0.01; Figure 1G).

Since these temporal dynamics in Cx43 amount did not align with changes in CV, Cx43 distribution at the cardiomyocyte borders was evaluated using immunofluorescence. Cx43 localization to the end location; that is, the ICD, slightly increased during development, reaching statistical significance compared to PD1 at PD40 (*p* < 0.05; Figure 1H). Notably, end‐localized Cx43 showed a significant positive correlation with CV_L_, while CV_T_ correlated with cardiomyocyte width (*p* < 0.05; Figure 1I,J). Additional morphometric data of the dataset are provided in Figure S2. Anisotropy ratio (AR) remained stable throughout the examined period (Figure 1K). In summary, both CV_L_ and CV_T_ increase during rat postnatal heart development, exhibiting distinct direction‐dependent maturation patterns. The Cx43 relocation to the ICD was a key determinant of CV maturation, with adult values achieved by PD20–30.

### 2.2 Abdominal Aortic Constriction at Postnatal Day 2 Resulted in a More Compensated Phenotype and Larger Left Ventricular Mass Than Constriction at Postnatal Day 6

**FIGURE 2:**
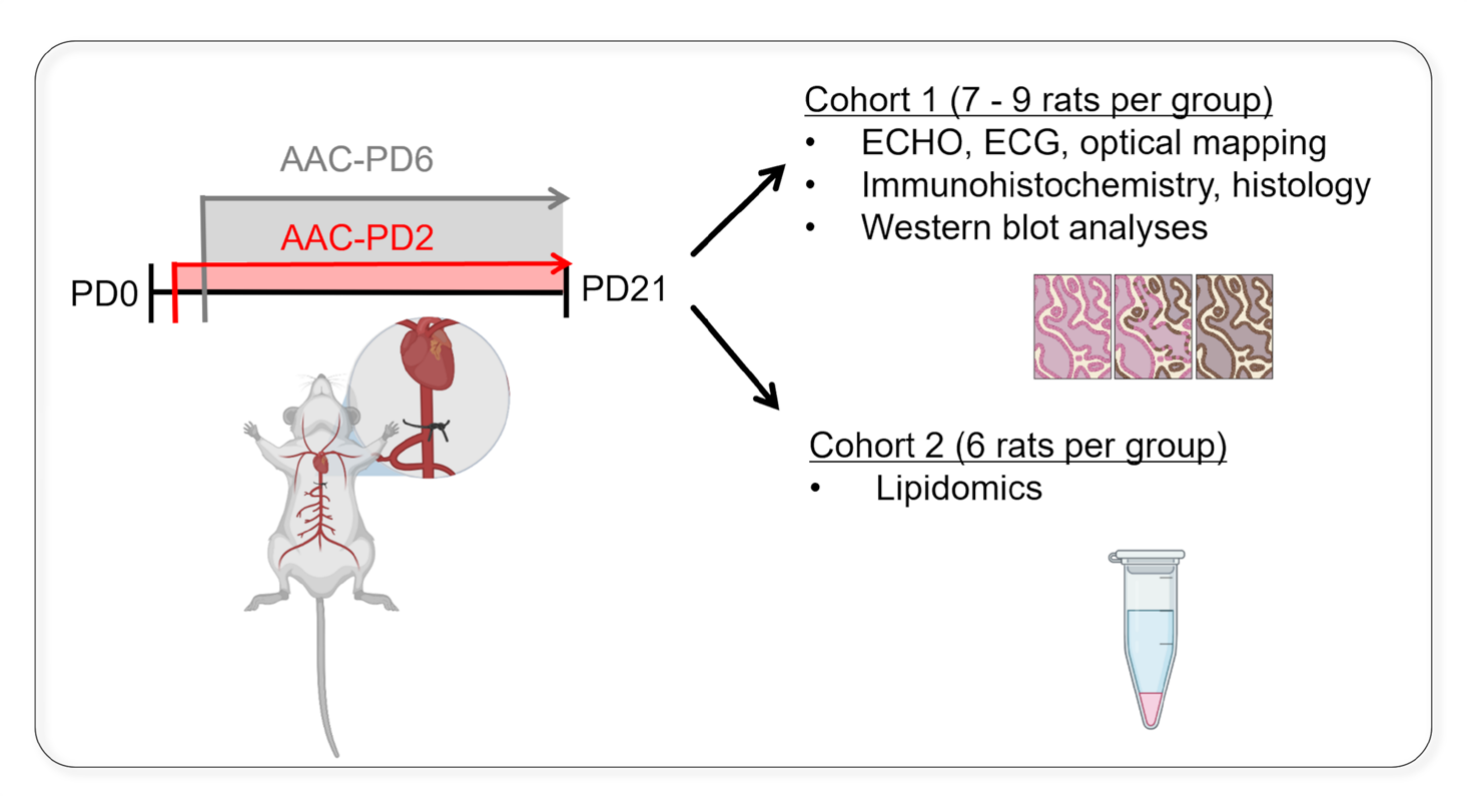
Experimental design of neonatal abdominal aortic constriction study. Schematic overview of the experimental timeline with surgery performed at postnatal day 2 (AAC‐PD2, abdominal aortic constriction at postnatal day 2—proliferative phase of cardiac development) or postnatal day 6 (AAC‐PD6, abdominal aortic constriction at postnatal day 6—non‐proliferative heart growth) and final analyses performed at postnatal day 21. Number of animals used for each analytical modality is given in parentheses.

**FIGURE 3:**
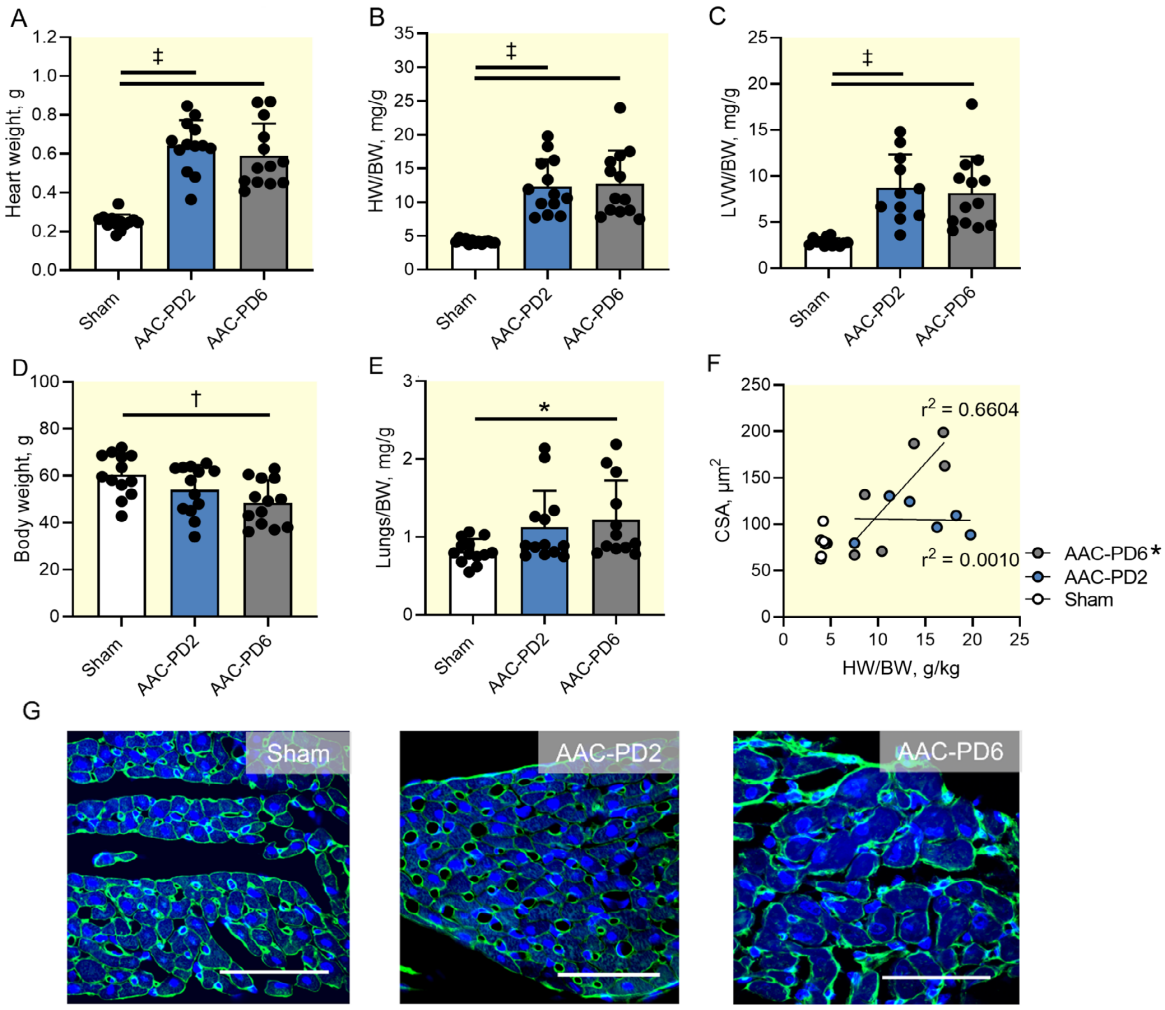
Higher level of phenotype compensation in AAC‐PD2. Quantification of the heart weight (A), heart weight normalized to body weight (HW/B; B), left ventricle weight normalized to body weight (LVW/BW; C), body weight (BW; D), and lung weight normalized to body weight (E) among experimental groups; *n* = 13 animals per group. Correlation analysis between HW/BW and cardiomyocyte cross section area (CSA) at the papillary muscle; *n* = 6 animals per group (F). Representative images of the cardiomyocyte cross section area all groups; wheat germ agglutinin staining of the myocyte membrane in green; nuclei stained by DAPI in blue (G). Scale bars represent 50 μm. Data are expressed as mean ± SD; One‐way ANOVA with Tukey multiple comparisons test was used for A–E, *p < 0.05, †*p* < 0.01, ‡*p* < 0.001. Pearson correlation coefficient was used for F, *p < 0.05.

Following the characterization of the increase in the CV_T_ and CV_L_ during physiological heart development, the changes induced by LVPO were assessed during the proliferative phase of cardiac growth (Figure 2). Neonatal pressure overload resulting from abdominal aorta narrowing (AAC surgery) led to a ~2.5‐fold increase in heart weight and a 3‐fold increase in the heart‐to‐body weight ratio (HW/BW) (*p* < 0.001 compared to Sham; Figure 3A,B). This increase was attributed to the enlargement of the LV in both AAC groups (*p* < 0.001 compared to Sham; Figure 3C). Body weight was significantly reduced in AAC‐PD6 (*p* < 0.01 compared to Sham), but not in AAC‐PD2 (Figure 3D). Lung‐to‐body weight ratio was also elevated in AAC‐PD6 only (*p* < 0.05 compared to Sham; Figure 3E). Both body weight and normalized lung weight are indicators of more cardiac compensation. Since in neonatal pressure overload–induced cardiomegaly most enlargement has been reported to result from an increased cross‐sectional area, with only a moderate contribution from increased cell length [41], we analyzed cardiomyocyte enlargement as cardiomyocyte cross‐sectional area (CSA). CSA from the horizontal section of the papillary muscle was significantly increased in the AAC‐PD6 (*p* < 0.05); however, CSA of the AAC‐PD2 was increased only slightly compared to Sham (Supporting Information, Figure S3). Since a similar increase in heart weight was observed in both AAC groups, the less pronounced cardiomyocyte enlargement of AAC‐PD2 indicates induced cell division after pressure overload imposed at the proliferative phase of cardiac development, as we reported in AAC‐PD2 earlier [39]. Moreover, the HW/BW significantly correlated with the CSA in the AAC‐PD6 (*p* < 0.05); however, it remains stable across the HW/BW in AAC‐PD2, indicating more intensive myocyte division in the more enlarged hearts (Figure 3F,G). The cardiomyocyte length determined from a circular layer of the LV free wall at the level of the papillary muscle, where cardiomyocytes are longitudinally oriented, showed similar size in all measured groups (67.00 ± 2.6 μm in Sham, 67.24 ± 3.0 μm in AAC‐PD2, and 69.14 ± 6.1 μm in AAC‐PD6). Additional cardiomyocyte morphometric parameters are summarized in the Table S1.

**FIGURE 4:**
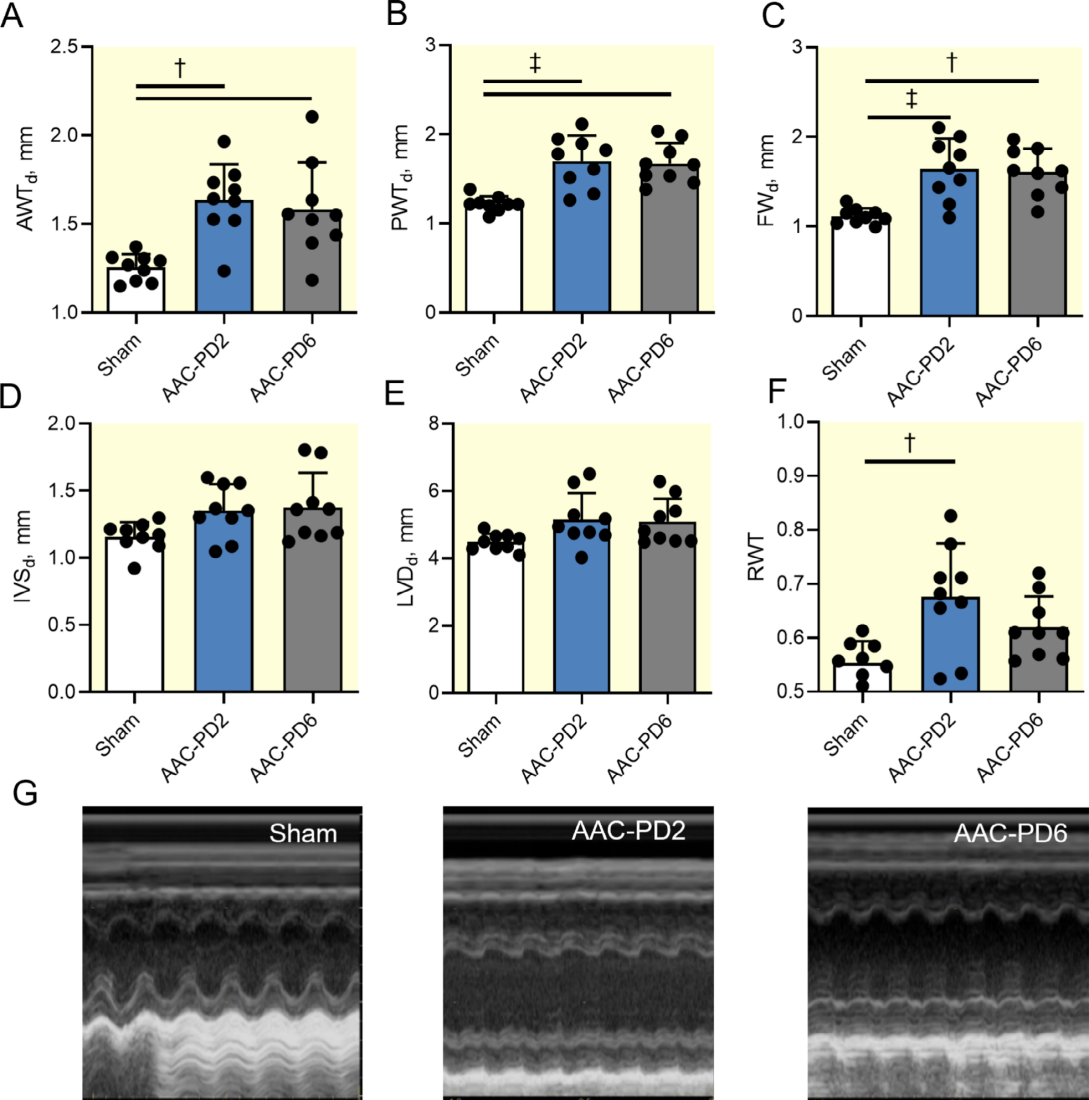
More pronounced increase of left ventricular mass in AAC‐PD2. Quantification of the echocardiographic measured left ventricular anterior (AWT_d_); *n* = 9 animals per group (A), posterior (PWT_d_); *n* = 9 animals per group (B), free wall (FW_d_); *n* = 9 animals per group (C), and interventricular septum (IVS_d_); *n* = 9 animals per group (D) wall thickness in diastole. Quantification of the diastolic left ventricular diameter (LVD_d_); *n* = 9 animals per group (E) and relative left ventricle thickness (RWT); *n* = 9 animals per group (F). Representative images of the M‐mode left ventricular echocardiography (G). Data are expressed as mean ± SD; one‐way ANOVA with Tukey multiple comparisons test, *p < 0.05, †*p* < 0.01, ‡*p* < 0.001.

Echocardiographic measurements showed a more pronounced increase in diastolic anterior wall thickness (AWTd) in AAC‐PD2 rats compared to the AAC‐PD6 group (*p* < 0.01 and *p* < 0.001 compared to Sham; Figure 4A). Thickness of the interventricular septum (IVS), posterior and free wall thickness (PWTd, FWd) did not show significant differences between AAC groups (Figure 4B–D). Similar increase in LV diastolic diameter (LVDd) in both AAC groups (Figure 4E). This fact together with different LV wall thickness elevation resulted in a significantly higher relative wall thickness (RWT) in AAC‐PD2 compared to Sham, but not in the AAC‐PD6 (*p* < 0.01; Figure 4F). These findings could correspond with enhanced wall thickening due to myocyte proliferation in the AAC‐PD2 rats.

In summary, AAC led to an increased heart mass in both groups, but AAC‐PD2 animals exhibited signs of more pronounced LV mass increase, which could correspond to the cardiomyocyte proliferation and sign of the more compensated phenotypic progression.

### 2.3 Similar In Vivo Functional Parameters in Both Abdominal Aortic Constricted Groups

**FIGURE 5:**
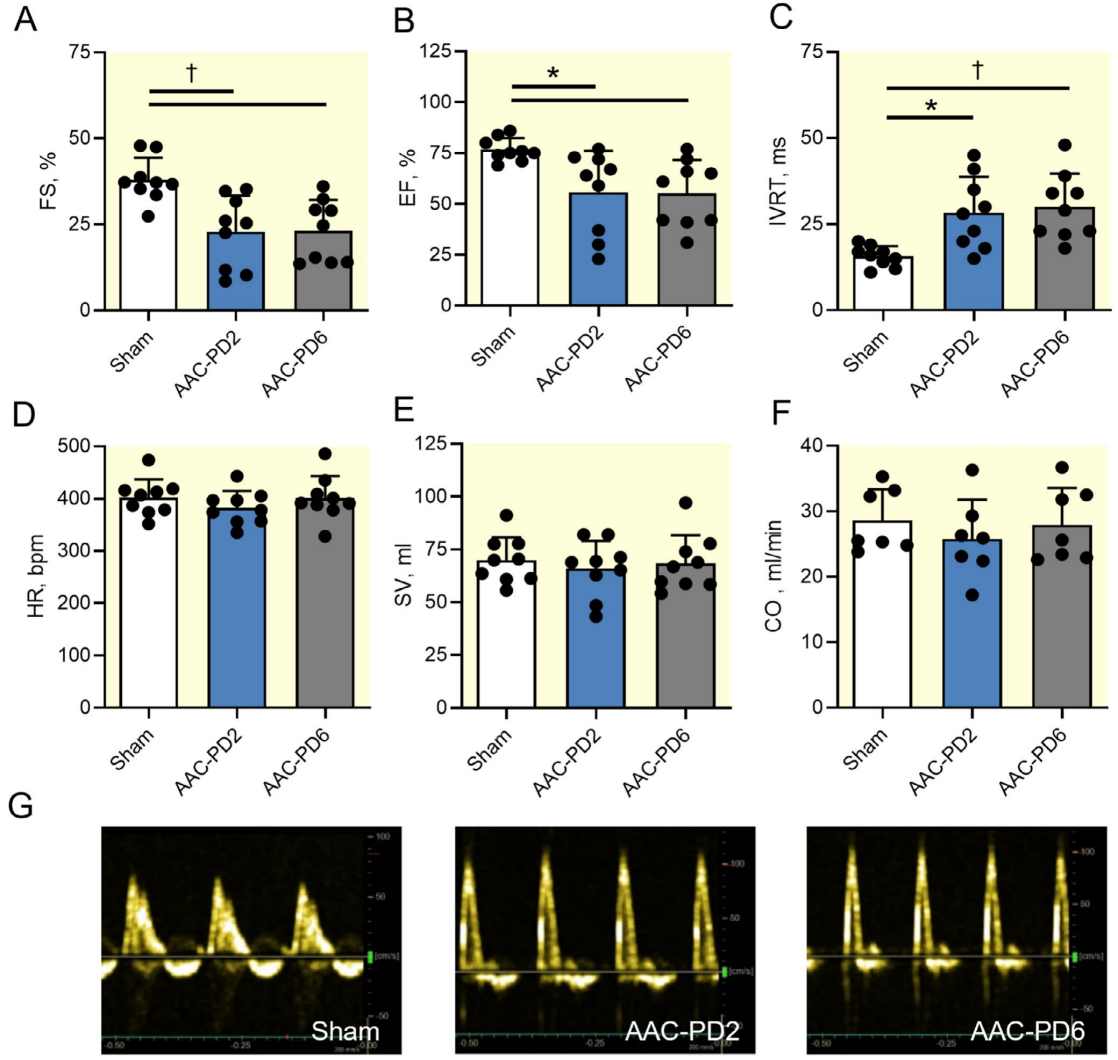
Similar echocardiographic parameters in both AAC groups. Quantification of the left ventricle fractional shortening (FS); *n* = 9 animals per group (A), ejection fraction (EF); *n* = 9 animals per group (B), isovolumetric relaxation time (IVRT); *n* = 9 animals per group (C), heart rate (HR); *n* = 9 animals per group (D), stroke volume (SV); *n* = 9 animals per group (E), and cardiac output (CO); *n* = 9 animals per group (F). Representative images of the flow across the mitral valve analyzed by Doppler measurement (G). Data are expressed as mean ± SD; one‐way ANOVA with Tukey multiple comparisons test, *p < 0.05, † *p* < 0.01.

Transthoracic echocardiography was used to functionally characterize phenotype progression. Systolic function expressed as fractional shortening (FS) and ejection fraction (EF) showed a similar reduction in both AAC groups (*p* < 0.05 and *p* < 0.01 compared to Sham; Figure 5A,B). Isovolumetric relaxation time (IVRT) values were increased in both AAC groups, suggesting comparable impairment of diastolic function (*p* < 0.05 and p < 0.01 compared to Sham; Figure 5C). Heart rate (HR), stroke volume (SV), and cardiac output (CO) were unchanged across groups (Figure 5D–F). Additional echocardiographic data showing the flow parameters at the mitral valve and pulmonary artery are provided in the Table S2.

Overall, echocardiography demonstrated no significant differences in systolic and diastolic function between both AAC groups.

### 2.4 Abdominal Aortic Constriction at Postnatal Day 2 Led to Preserved Conduction Properties

**FIGURE 6:**
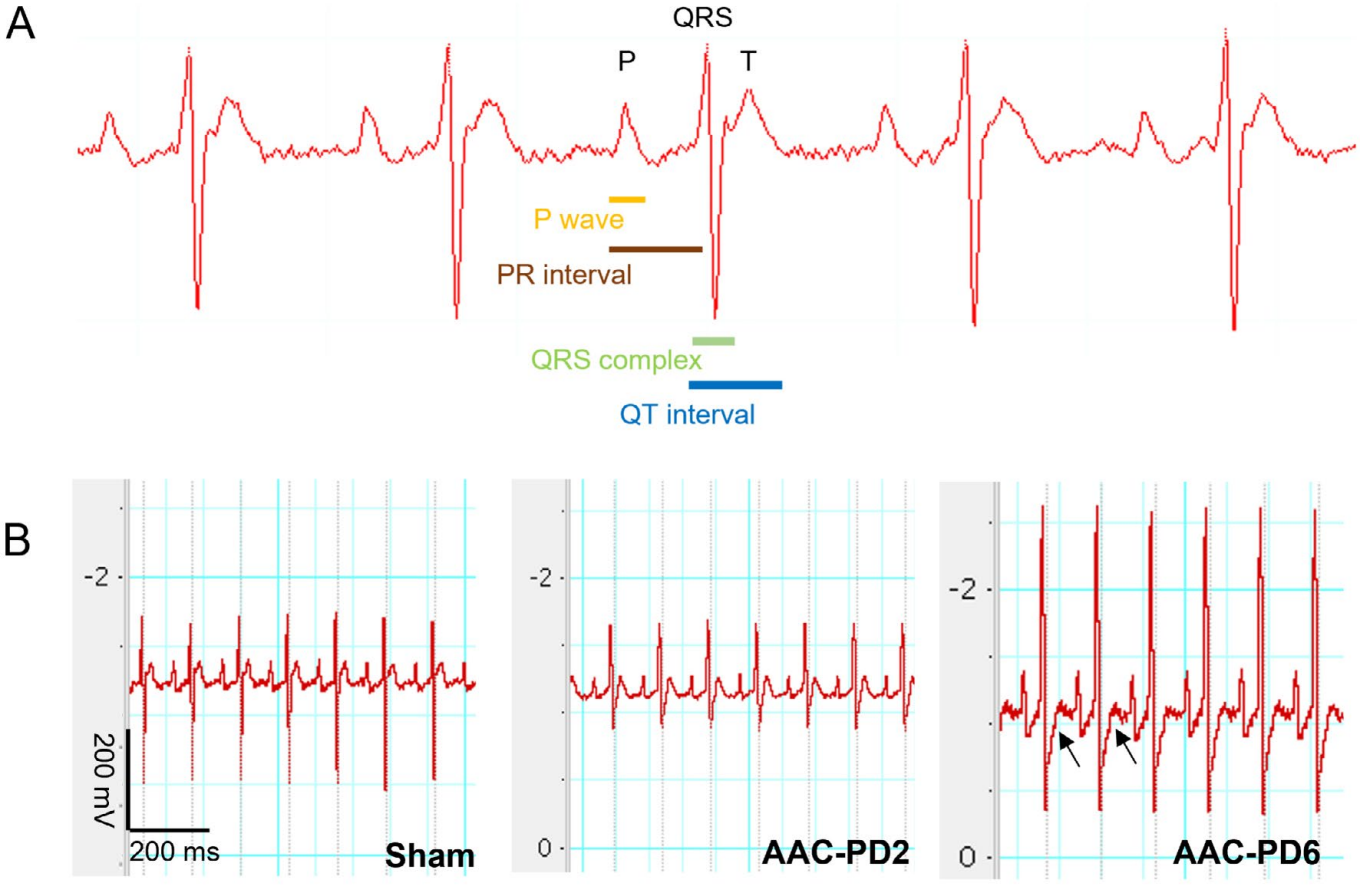
Preserved electrical characteristics in AAC‐PD2 assessed by electrocardiography. Representative electrocardiogram with depicted waves and intervals (A). Representative electrocardiogram for all groups (ST depression indicated by the arrows) (B).

Electrocardiographic (ECG) analysis (Figure 6A) revealed significant QRS prolongation in the AAC‐PD6 but not AAC‐PD2 (*p* < 0.05 compared to Sham; Table 1). There were no significant changes in other QT and QTc, indicating unaltered repolarization. Atrioventricular conduction, expressed as PR interval, did not reveal any significant differences among the groups. HR showed a mild decrease in AAC‐PD2 (5% relative to Sham) and a more pronounced reduction in AAC‐PD6 (10% relative to Sham) (Table 1). ST segment depression, a marker of myocardial hypoxia, was observed in AAC‐PD6 only (Figure 6B).

**TABLE 1:** ECG parameters of the AAC animals.

Parameter	Sham	AAC‐PD2	AAC‐PD6
P wave, ms	15.1 ± 2	18.6 ± 5	13.1 ± 1
QRS, ms	17.7 ± 2	20.2 ± 4	24.0 ± 3*
QT, ms	47.0 ± 8	53.8 ± 12	53.6 ± 7
QTc, ms	127.8 ± 18	146.9 ± 34	138.0 ± 16
HR, bpm	448 ± 37	427 ± 35	408 ± 50

*Note:* Data are expressed as mean ± SD; one‐way ANOVA with Tukey multiple comparisons test.

* *p* < 0.05 vs. Sham.

Given that prolonged conduction through ventricles is an important pro‐arrhythmogenic feature associated with LVH, we aimed to determine whether increased QRS duration is caused by the altered conduction due to excessive fibrosis, conduction slowing, or simple LV enlargement. The level of myocardial fibrosis determined by Picrosirius Red (PSR) staining was rather decreased in both AAC groups but did not reach statistical significance (1.18 ± 0.2 PSR positivity/cardiomyocytes for Sham, 0.65 ± 0.2 for AAC‐PD2, and 0.67 ± 0.6 for AAC‐PD6, Figure S4).

**FIGURE 7:**
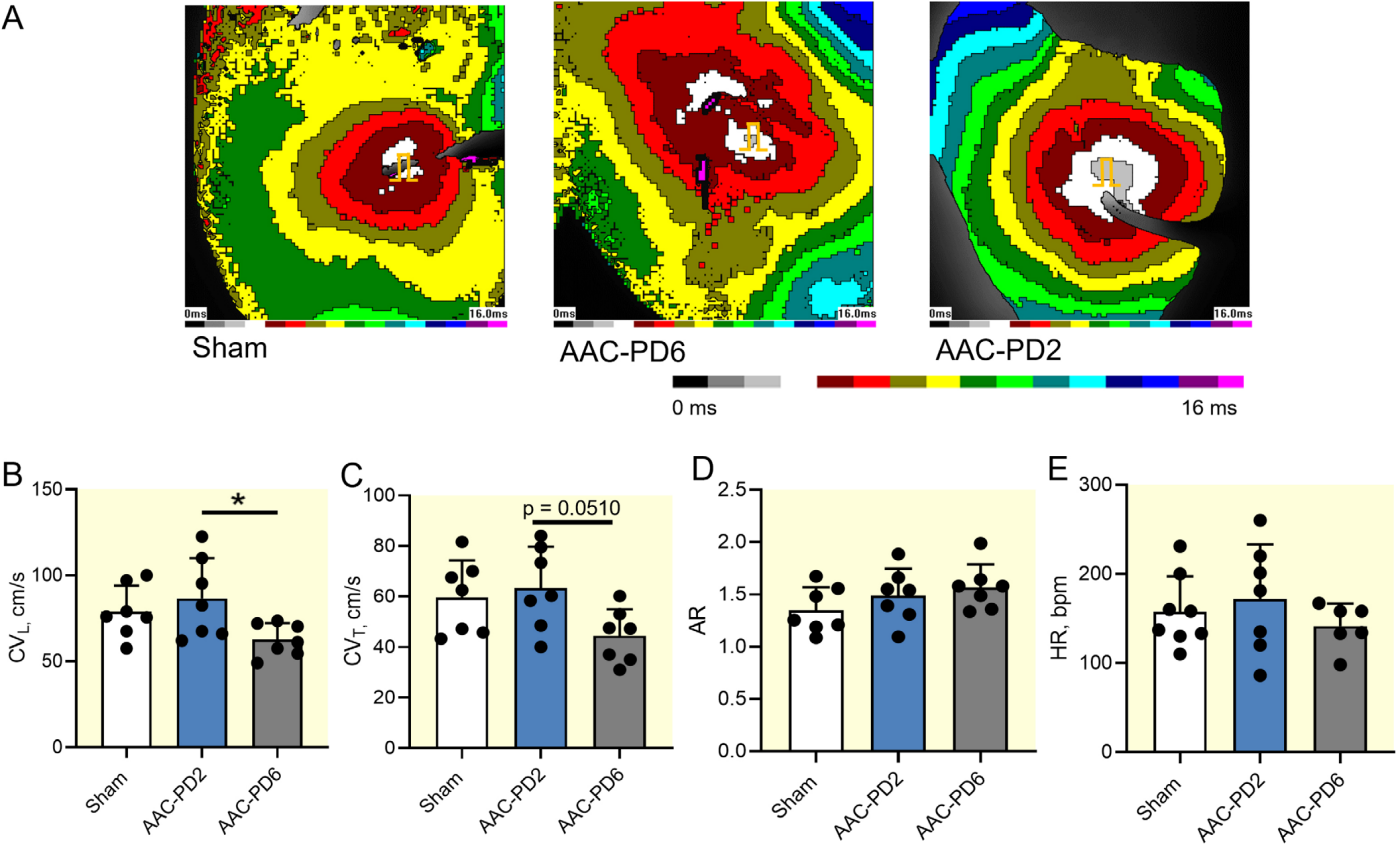
Preserved epicardial conduction velocity in AAC‐PD2. Representative epicardial maps (stimulation from the center of the left ventricle (color band represent 1 ms interval)) (A). Quantification of the conduction velocity in the longitudinal direction (CV_L_); *n* = 7 animals per group (B), transversal direction (CV_T_); *n* = 7 animals per group (C). Quantification of the anisotropy ratio (AR); *n* = 7 animals per group (D), and ex vivo measured heart rate (HR); *n* = 6–7 animals per group (E). Data are expressed as mean ± SD; one‐way ANOVA with Tukey multiple comparisons test, *p < 0.05.

Optical mapping revealed a significantly reduced CV_L_ in AAC‐PD6 compared to AAC‐PD2 (*p* < 0.05) and a similar trend for the CV_T_ (*p* = 0.0510). Despite an obvious trend in CV slowing at AAC‐PD6 compared to Sham, the changes did not reach statistical significance. On the other hand, AAC‐PD2 showed no changes in both CV_L_ and CV_T_ when compared to the Sham operated controls (Figure 7A–C). AR did not differ between groups, and ex vivo HR showed a trend to the reduction in AAC‐PD6 but not in AAC‐PD2 animals (Figure 7D,E). Notably, the CV decrease did not correlate with functional parameters (Figure S5).

Since voltage‐gated sodium channels play an important role in proper CV, we assessed their function by analyzing the dF/dt_max_ from the optical recordings. We did not observe significant differences among the groups (0.34 ± 0.34 units/ms for Sham, 0.27 ± 0.25 units/ms for AAC‐PD2, and 0.28 ± 0.30 units/ms for AAC‐PD6). Action potential duration at 50% and 90% repolarization (APD_50_ and APD_90_) did not significantly differ among the groups (APD_50_ was 55.83 ± 13.0 ms for Sham, 60.25 ± 13.0 ms for AAC‐PD2, and 60.10 ± 6.2 ms for AAC‐PD6; APD_90_ was 93.00 ± 9.5 ms for Sham, 92.50 ± 9.0 ms for AAC‐PD2, and 96.83 ± 14.8 ms for AAC‐PD6). These findings are in line with the unaltered ECG parameters describing repolarization.

Electrophysiological data together indicate preserved conduction properties in the AAC‐PD2, while the AAC‐PD6 animals showed conduction slowing typical of adverse LVPO electrical remodeling. The structural background of the reduced CV in AAC‐PD6 is not likely connected to the altered function of ion channels.

### 2.5 Structural Changes in Neonatal Rat Hearts After Abdominal Aortic Constriction at Postnatal Day 2

**FIGURE 8:**
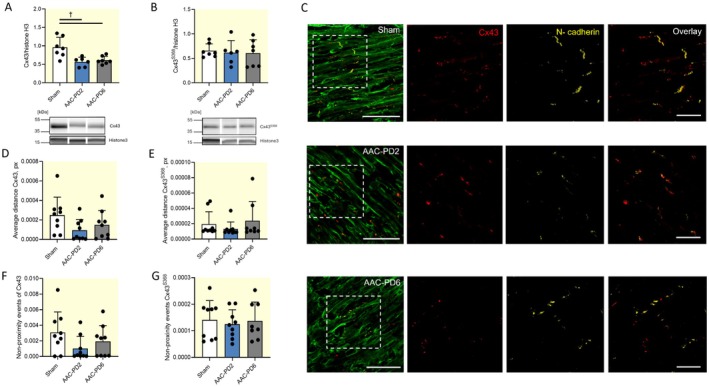
Higher Cx43 and Cx43^S368^ localization to the intercalated disks in AAC‐PD2. Protein expression of Cx43 and Cx43^S368^ in left ventricle normalized to histone H3; *n* = 6–7 animals per group (A, B). Representative images of the left ventricle free wall circular layer at the level of the papillary muscle showing localization of Cx43 (red) in relation to the N‐cadherin (marker ICD; yellow); wheat germ agglutinin staining of the myocyte membrane in green (C). Quantification of the average distance between N‐cadherin the for Cx43 and Cx43^S368^; *n* = 8–9 animals per group, 10 ROI per animals (D, E) and non‐proximity event between N‐cadherin for Cx43 and Cx43^S368^; *n* = 8–9 animals per group, 10 ROI per animals (F, G). Scale bar 50 μm for full image and 25 μm for inset. One‐way ANOVA with Tukey multiple comparisons test; †*p* < 0.01.

Cx43 and its phosphorylated form Cx43^S368^ are the principal gap junction proteins in the ventricular myocardium that determine CV. The main focus of the next series of studies was on both forms of Cx43 because of their essential role in cardiac electrical conduction [42]. Despite the decrease in Cx43 total expression in both AAC groups compared to Sham (*p* < 0.01), we did not observe differences either in Cx43 and Cx43^S368^ amounts between AAC‐PD2 and AAC‐PD6 that would explain the decreased CV in AAC‐PD6 (Figure 8A,B; Figures S6 and S7). Therefore, we next addressed Cx43 and Cx43^S368^ localization within the ventricular tissue. Double immunostaining of Cx43 and Cx43^S368^ with the ICD marker (N‐cadherin) in the circular layer from the LV free wall at the level of the papillary muscle where cardiomyocytes are longitudinally oriented showed an increased level of Cx43 localization in proximity to the ICD in both AAC groups (Figure 8C). Proximity analysis performed in the Fiji Distance analysis plugin revealed a trend to decrease in both Cx43 and Cx43^S368^ distance to N‐cadherin in AAC‐PD2 compared to Sham; however, it did not reach statistical significance, mostly due to higher variability (Figure 8D,E). Similarly, the amount of the non‐proximate Cx43 and Cx43^S368^ was lowest in the AAC‐PD2 rats; however, it did not reach statistical significance compared to Sham (Figure 8F,G).

### 2.6 Lipidomic Analysis of the Neonatal Rat After Abdominal Aortic Constriction at Postnatal Day 2

**FIGURE 9:**
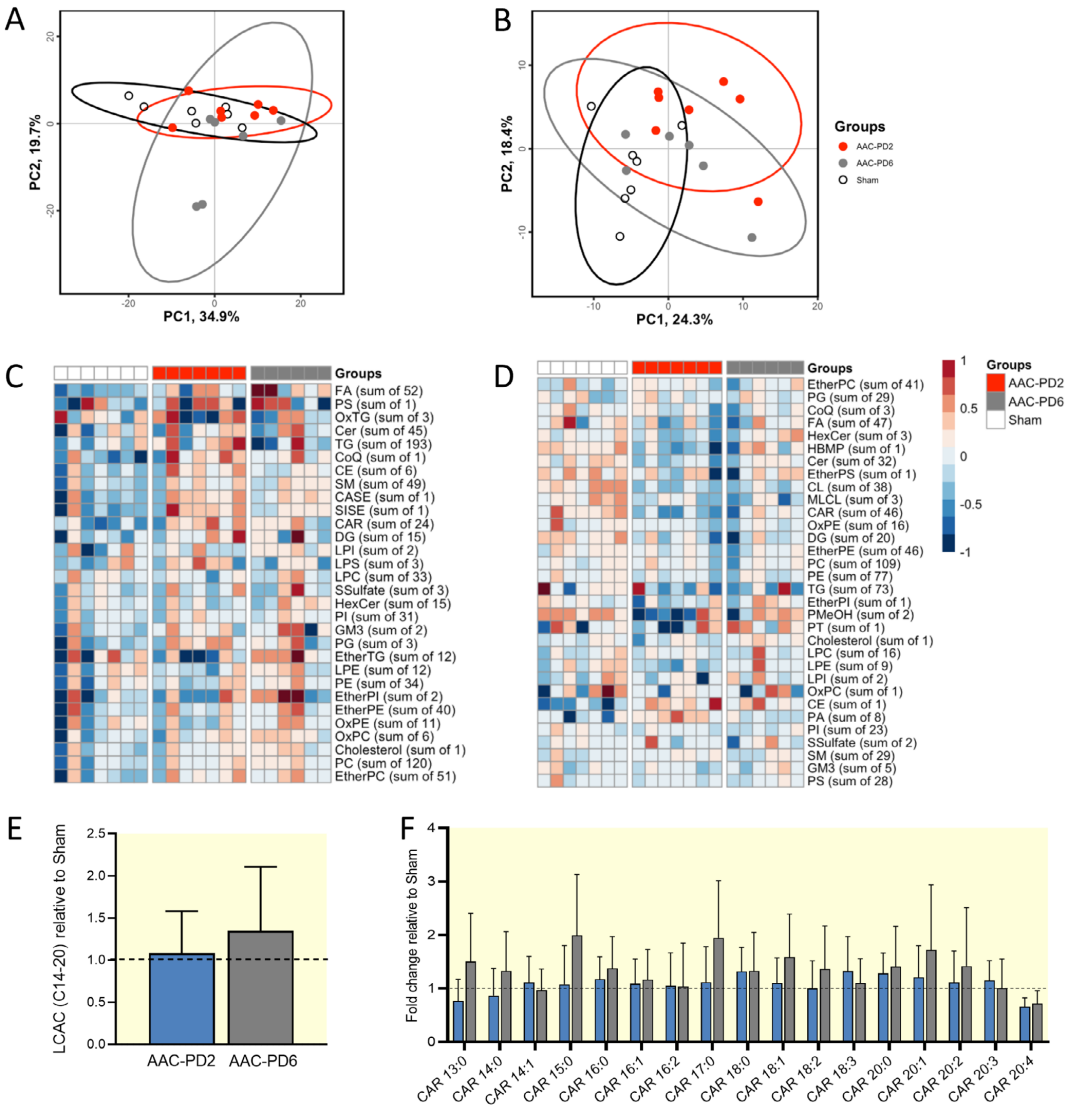
Lipidomic alterations in the AAC‐PD2 phenotype. Principal component analysis of the lipids in plasma; *n* = 6 animals per group (A) and myocardium; *n* = 6 animals per group (B). Heat maps of the lipid clusters for the plasma; *n* = 6 animals per group (C) and myocardium; *n* = 6 animals per group (D). Total level of the long chain acylcarnitines; *n* = 6 animals per group (LCACs) (E) and their specification according to the carbon atom numbers; *n* = 6 animals per group (F). One‐way ANOVA with Tukey multiple comparisons test.

To explore the contribution of the altered lipid spectrum previously reported in AAC‐PD2 rats [38] to the differential conduction phenotypes, we assessed lipidomic profiles in plasma and LV myocardium, focusing on lipids implicated in adverse electrical remodeling. Principal component analysis (PCA) did not reveal clear separation among the groups in plasma and heart tissue (Figure 9A,B). In total, 712 and 715 lipids were detected for plasma and LV myocardium, respectively. Alterations in lipid clusters in plasma and myocardium are visualized in the heatmaps (Figure 9C,D). Focusing on the long‐chain acylcarnitines (LCACs; C14‐20) [43] with known adverse effects on electrophysiology [44, 45], the increase in comparison to the Sham was observed in the AAC‐PD6, but not in the AAC‐PD2 (Figure 9E). Specifically, C18:1 acylcarnitine levels were increased by 58% in the AAC‐PD6 compared to Sham, while the AAC‐PD2 showed only a 10% increase (Figure 9F); however it did not reach statistical significance, mostly due to higher variability of the data.

Altogether, preserved CV in AAC‐PD2 compared to Sham was accompanied by the trend of LCAC accumulation and the tendency to enhanced Cx43 localization at the ICD.

## 3 Discussion

Cardiac pressure overload in adults is associated with adverse electrical remodeling leading to an increased risk of arrhythmias. Progression of this phenotype has been linked to alterations in Cx43 as the main gap junction protein in the ventricular myocardium [11, 17, 46, 47]. Electrical remodeling is attenuated when the pressure stimulus is applied in the proliferative phase of postnatal cardiac development. While the CV_L_ displayed the expected slowing in the AAC‐PD6 hearts, CV was unaltered in the AAC‐PD2 animals. This phenotype was accompanied by a trend toward increased Cx43 and Cx43^S368^ localization at ICD and higher stability of LCACs levels.

During the neonatal period, the heart undergoes important adaptations to accommodate the hemodynamic transition in postnatal life [48]. In this highly plastic stage, the heart responds differently to pathological stimuli [49, 50]. Regenerative healing, rather than scar formation, has been observed after injury during the first postnatal days [30, 31, 32, 51]. However, this capacity is quickly lost, likely due to the cessation of cardiomyocyte proliferation around PD4 in rodents [5, 31]. Notably, the level of proliferation could be increased under the pressure challenge in both left and right ventricles [29, 39, 52]. Absence of the M2 macrophages led to the suggestion that proliferation under pathological conditions could be a cell‐autonomous response of neonatal cardiomyocytes [52]. We recently demonstrated that LVPO at PD2 leads to long‐term reprogramming of cardiac structure and function, with distinct involvement of molecular pathways and cell death mechanisms compared to post‐myocardial infarction‐induced cardiomegaly [53].

The current study provides the first comprehensive functional characterization of the AAC‐PD2 model by echocardiography. While previous studies reported on blood pressure and contractility in adult AAC‐PD2 animals during acute aortic ligation [37], echocardiographic evaluation, as a gold standard for the functional assessment, was lacking. In comparison with AAC‐PD6 animals, the AAC‐PD2 animals exhibited an increased LV wall thickening accompanied by unchanged LV diameters, resulting in the higher LV mass in AAC‐PD2 rats. These findings, together with only slightly increased cardiomyocyte CSA, support the myocyte proliferation as one of the mechanisms contributing to the observed cardiac enlargement, as was previously demonstrated at AAC‐PD2 animals with a similar HW/BW ratio [39]. Systolic and diastolic parameters of LV function were similar in both AAC groups.

Given that higher mortality in LVPO patients is attributed mainly to the higher propensity of malignant arrhythmias [13], it is important to study the electrical remodeling of the heart with pressure overloaded applied at the proliferative phase of cardiac development. Despite extensive research mapping the electrophysiology of various genetic abnormalities [54, 55, 56] or aged myocardium [57, 58], little is known about the structural and electrophysiological maturation process. Structural development was better characterized, showing proportional increases in cardiomyocyte length and width accompanied by a 10‐fold increase in LV mass and an 11‐fold increase in fiber volume between rat PD5 and PD125 [59, 60, 61, 62]. In contrast, electrophysiological development during the early postnatal period remains far less understood. Prior work in canine and guinea pig hearts reported ~40%–60% increases in CV between neonatal and adult stages [63, 64] and changes in action potential duration, ion channel expression, and calcium handling [63, 65, 66, 67]. However, comprehensive data on the development of conduction characteristics during postnatal rat growth remained unknown. In the context of Cx43 maturation, studies in neonatal rodent myocardium have shown widespread localization of Cx43 at the cardiomyocyte surface, but did not assess functional characteristics [33]. The current data provide detailed insight into rat heart maturation, showing stepwise increases in CV_L_ and continuous increases in CV_T_, with adult values reached at PD20 and PD30.

Since at the PD21, the binucleation of the rat cardiomyocytes is also completed [68], we assessed the pressure‐induced changes at this age. ECG recordings showed QRS prolongation in AAC‐PD6 but not in AAC‐PD2. As myocardial fibrosis levels were not significantly different between groups, we focused on epicardial conduction. Optical mapping confirmed preserved CV_L_ and CV_T_ in AAC‐PD2, whereas AAC‐PD6 hearts showed significant slowing. Notably, CV reduction did not correlate with functional parameters, consistent with previous findings in human and murine LVPO models where arrhythmia susceptibility was independent from systolic dysfunction [11].

In adult rodent models, CV slowing in pressure LVPO is a hallmark of pro‐arrhythmogenic remodeling [11, 17]. Asymmetric conduction slowing increases the likelihood of re‐entry by shortening the circulating path length [12, 16, 69]. CV slowing was mainly connected to Cx43 expression or distribution, the function of the voltage‐gated sodium channels, and to myocardial fibrosis. In contrast to atrial tissue, where fibrosis is critical for electrical remodeling [70], ventricular conduction is more sensitive to Cx43 alterations. During LVPO and heart failure progression, Cx43 lateralization or heterogeneous distribution has been associated with electrical remodeling in these cardiomyopathies [19, 71, 72, 73, 74]. Non‐ICD Cx43 localization has been linked to higher arrhythmia risk, even in the absence of changes in its expression [11, 17, 75]. In a canine model of HF, Cx43 remodeling was shown to occur prior to structural changes, in the absence of fibrosis, and described as critical in slowed CV and ventricular arrhythmia development [47].

The results revealed no significant differences in total Cx43 or Cx43^S368^ expression between AAC groups. Maximal dF/dt from the action potential upstroke and PSR staining showed similar values among the AAC groups, suggesting that there are no substantial differences in voltage‐gated sodium channel function or myocardial fibrosis that would affect CV. However, it should be noted that there is no full agreement regarding the relationship between Cx43 gap junction channel function and action potential upstroke velocity. Some studies have suggested that inhibition of gap junctions led to preserved or even increased AP upstroke velocity despite decreased CV [76, 77, 78], whereas others reported the opposite findings [79, 80, 81]. Although patch‐clamp measurements would be necessary to definitively assess voltage‐gated sodium channel function in AAC animals, our data do not indicate any functional alterations. Unlike the total expression of Cx43 and Cx43^S368^, they both displayed a trend toward higher localization close to ICD in the AAC‐PD2 hearts compared to the AAC‐PD6. This finding is consistent with preserved conduction parameters observed in AAC‐PD2 rats and was reported as an important factor in the decreased arrhythmias vulnerability [11, 17].

Despite the fact that we analyzed only neonatal Wistar rats, strain dependence in pressure overload phenotype progression has been extensively discussed in adult rodents [82]. While rats represent only 5% of transverse aortic constriction (TAC) studies, as reported in a recent meta‐analysis, most knowledge on strain dependence is based on mice [82]. Among hemodynamic overload rat studies, the work by Sykora et al. focuses on the role of the strain in the phenotype progression. They have shown no differences at the Cx43 level between normotensive and hypertensive rat strains [83]. Interestingly, right ventricular failure following pulmonary trunk banding in rats seems to be more strain dependent, with the favorable outcomes observed in Fischer344 rats compared to Wistar and Sprague Dawley rats [84]. In mice, BALB/c strain has been shown to develop progressive ventricular dilation due to TAC with pulmonary congestion and higher mortality compared to C57BL/6J mice [85]. On the other hand, mutation in the nicotinamide nucleotide transhydrogenase gene in C57BL/6J mice has protected mice from oxidative stress and heart failure after TAC compared to the inbred C57BL/6N strain [86]. In the context of strain dependence in pressure overloaded hearts, it should be noted that evidence is based on adult animals and its impact on the neonatal heart remains to be further addressed. This is particularly important since neonatal pressure overload has generally been reported to produce a phenotype with decreased levels of myocardial fibrosis [29, 37] which could significantly influence the final phenotype.

A concerted rise in multiple lipids during phenotype progression in various pressure‐overload models, including AAC‐PD2 [87] has been reported. In adipose triacylglycerol hydrolysis mediated by adipose triglyceride lipase (ATGL) knockdown mice, perturbation of adipose tissue lipolysis by ATGL deficiency ameliorated pressure‐induced heart failure, demonstrating the importance of cardiac lipidome changes in TAC progression [88, 89, 90]. Additional studies have confirmed the harmful effects of lipidomic spectrum alterations [91, 92, 93]. Notably, these adverse impacts of altered lipidome appear not to be restricted to the LV or to rodents, as similar alterations have been described in the pressure‐overloaded right ventricle, as well as in large animal models and humans [91, 92, 94]. Given the emerging evidence that lipids also contribute to adverse electrical phenotype [44], a lipidomic analysis was performed to gain further insight into the preserved conduction properties in AAC‐PD2. Higher levels of medium‐ and long‐chain acylcarnitines have been implicated in cardiovascular disease and arrhythmia risk [93]. Further studies have shown that C16 and C18:1 acylcarnitines impair electrophysiological properties and cause conduction deficits in isolated rat hearts and cardiomyocyte cultures [45, 95, 96]. In our study, we found a trend toward elevated LCAC levels in AACPD6, while these levels were preserved in AACPD2. We have also identified a specific elevation of the C18:1 acylcarnitine in AAC‐PD6, while remaining stable in AAC‐PD2 compared to Sham‐operated animals. These changes could contribute to the decreases in CV observed in AAC‐PD6 animals.

The current study provides a characterization of electrophysiological development under both physiological and elevated afterload conditions. Despite differences in ion channels' contributions to the action potential between specific heart chambers in rodents, larger animal models, and humans [97, 98], the overall pattern of electrophysiological maturation is conserved. Widespread Cx43 distribution in neonatal ventricular myocytes has been described in both rodents and humans [33, 99]. Similarly, expression of Cx43 across both ventricles and atria is well conserved among mammals [100, 101]. The electrophysiological response to pressure stimulus also appears to be conserved across mammalian species, with Cx43 downregulation, dephosphorylation, and lateralization described in various animal models as well as in human patients [11, 17, 19, 102]. With respect to myocyte proliferation [103, 104] the transition from hyperplastic to hypertrophic growth occurring within the first postnatal days in rodents [5, 31, 105] is placed also around days 1–2 in the neonatal pigs [106, 107]. In humans, although only limited data are available, Mollova et al. reported that, in addition to hypertrophy, cardiomyocyte number in the LV increases until approximately 20 years of age [6], potentially conferring greater regenerative potential. A case report of naturally occurring neonatal infarction [51] with complete functional recovery, as well as a study of 14 patients (median age 0.7 years) operated for anomalous left coronary artery from the pulmonary artery [108] showing minimal scarring, suggests a privileged capacity of the human neonatal heart to tolerate insults that would cause severe, irreparable damage in the adult. This capacity is likely related to higher proliferative and regenerative potential. Thus, the different reactions to pressure overload in proliferative versus nonproliferative heart growth observed in the current study are consistent with these findings, supporting the relevance of the neonatal rat model for larger mammals, including humans.

## 4 Methods

### 4.1 Ethics Statement

The study was conducted in accordance with the Guide for the Care and Use of Laboratory Animals (published by the National Academy of Sciences, National Academy Press, Washington, D.C.). Experimental protocols were approved by the Animal Care and Use Committee of the Institute of Physiology, the Czech Academy of Sciences, and the Institute of Anatomy, First Faculty of Medicine, Charles University (Permit Number: 75/2021).

### 4.2 Animals

Wistar rats were used to investigate conduction properties and the underlying mechanisms throughout postnatal development. Specifically, PD 1, 4, 6, 11, 20, 30, 40, 60, and 90 were selected for analysis. At each time point, at least five animals were analyzed using optical mapping, followed by histological and immunohistochemical characterization. Both sexes were included up to PD 20. From PD 20 onward, only males were used.

### 4.3 Model of the Neonatal Pressure Overload

Pressure overload was induced by AAC in neonatal Wistar rats of both sexes, as we described before [38, 39]. Briefly, the abdominal aorta was exposed and constricted using the 0.25 template. Surgery was performed in the proliferative phase of the postnatal cardiac growth; that is, PD2 (AAC‐PD2). Animals underwent AAC at PD6 (AAC‐PD6, resulting in a purely hypertrophic response [38]), and PD2 sham‐operated animals were used as controls. Animals were assigned to the final analysis groups on PD21, when the phenotype is fully established [39] (Figure 2).

### 4.4 Phenotype Progression Analyses by Echocardiography and Morphology

Prior to final analyses on PD21, cardiac geometry and function were assessed using GE Vivid 7 Dimension (GE Vingmed Ultrasound, Horten, Norway) and 12 MHz linear matrix probe ML12L. Animals were anesthetized with 2% isoflurane (Forane, Abbott Laboratories) mixed with room air, placed on a heating pad, and their rectal temperature was maintained between 36.0°C and 37.5°C. The following parameters of LV geometry were assessed: anterior, posterior, free wall, and interventricular septum thickness in both systole and diastole (AWTs, AWTd, FWTs, FWTd, PWTs, PWTd, IVSs, IVSd) and left ventricular diameters in systole and diastole (LVDs, LVDd). Doppler pulse wave measurements were used to assess blood flow in the pulmonary artery and at the level of the mitral annulus [109, 110]. Based on LV geometry, the RWT, FS, SV, and EF, CO were derived (exact equations are provided in the extended methods within the Supporting Information).

Phenotype progression was further validated using morphological parameters, specifically HW/BW, LVW/BW, and lung/BW.

### 4.5 Electrocardiography Measurement and Analysis

At PD21, surface ECGs were recorded in anesthetized rats (pentobarbital, 60 mg/kg, i.p.) using subcutaneous 23‐gauge needle electrodes placed on each limb. ECG signals were acquired with the PowerLab system (ADInstruments) and analyzed using LabChart Pro software. The following parameters were evaluated from the averaged signal (lead II equivalent): RR interval, P wave duration, QRS complex duration, QT interval, and corrected QT interval (QTc) [111, 112].

### 4.6 Epicardial Conduction Velocity Assessment

Conduction parameters and activation patterns were assessed by optical mapping as previously described [113, 114]. Hearts were isolated from anesthetized (pentobarbital, 60 mg/kg, i.p., Bioveta, Czech Republic) and heparinized (1000 IU/kg, i.p., Zentiva, Czech Republic) rats and transferred to ice‐cold Tyrode solution (in mmol/L: 145 NaCl, 5.9 KCl, 1.1 CaCl_2_, 1.2 MgCl_2_, 11 glucose, 5 HEPES, pH 7.4, Penta Chemicals). The aorta was cannulated, and the heart was perfused with oxygenated Tyrode solution (37°C, 100% O_2_). For PD1–PD11 hearts, cannulation was performed under a dissection microscope (Olympus SZX12) to ensure precision. Since the correct cannulation is crucial for adequate perfusion and unaffected electrical activity [114], we have paid special attention to the establishment of normal heart rhythm, especially in the earliest developmental stages (the LV length of the PD1 is 5 mm, aortic width ~0.3 mm), proving the adequate perfusion. This was further confirmed by the presence of the matured activation pattern (Supporting Information, Figure S8).

After 5–10 min of stabilization and adequate perfusion assessment, LV was placed parallel to the optical system. Electrical activity was mapped using voltage‐sensitive dye di‐4‐ANNEPS and motion inhibitor blebbistatin (200 μL of 0.125% and 50 μL of 0.417%, respectively) [115, 116]. Imaging was performed under the stimulated rhythm (from the same position in the center of the LV—since CV is dependent on fiber orientation [117]) using a bipolar atraumatic electrode; 250 ms cycle length; 5 mA intensity; 2 ms pulse duration (Figure S1). Acquisition was performed with a high‐speed ULTIMA L camera (SciMedia, Japan) attached to the THT Mesoscope (Brain Vision Inc., Japan) with tandem Leica optics. The system was fitted with a high‐intensity LEX3 LED Light Source (Brain Vision Inc., Japan) [118]. Data were analyzed using BV_analyzer (Brain Vision Inc., Japan) [119] to evaluate activation patterns, CV_L_, CV_T_. CV_L_, CV_T_ were measured from the paced rhythm at the same pacing cycle length since their duration affects the CV [120]. CV was assessed in the context of the fiber orientation with CV_L_ defined as propagation parallel to fibers and CV_T_ as propagation perpendicular to them [21]. AR was defined as CV_L_/CV_T_ [109]. Normalized maximum differential value of the optical action potential upstroke (dF/dt_max_) and APD_50_ and APD_90_ were analyzed using BV_analyzer (Brain Vision Inc., Japan) as described earlier [21, 121].

After optical mapping, hearts were fixed in 4% paraformaldehyde and embedded in paraffin for further analysis.

### 4.7 Western Blot Analyses

For assessment of the Cx43 changes related to the postnatal maturation of the conduction velocity, samples were homogenized and fractionated to separate homogenate, non‐junctional, and junctional fractions of Cx43 [122]. Briefly, 20 μg of total protein were separated on precast SDS‐PAGE gels (Bio‐Rad, USA) using a Mini‐PROTEAN Tetra Vertical Electrophoresis Cell (Bio‐Rad). Proteins were transferred to Immobilon‐FL PVDF Membranes (IPFL00010, Merck) using a Trans‐Blot Turbo Transfer System (Bio‐Rad, USA). Membranes were blocked in 5% fat‐free milk (Sigma) in 0.1% Tween 20 in tris buffered saline (TTBS) for 1 h. Membranes were then incubated with anti‐Cx43 rabbit primary antibody (Sigma # C6219) in 1% fat‐free milk in TTBS overnight at 4°C. After three 5‐min washes with fresh TTBS, membranes were incubated with a goat anti‐rabbit secondary antibody (Bio‐Rad #1706515) in 1% milk in TTBS for 1 h. The membranes were then washed 3 times in TTBS and incubated with SuperSignal West Femto Maximum Sensitivity Substrate solution (Thermo Fisher). Total protein detection was performed by immersing the membranes into Ponceau S solution for 4 min on a shaker and subsequently immersing them in water for 1 min with shaking. Detection was performed in a Chemidoc Imaging System (Bio‐Rad), and image analysis was performed using ImageLab 5.1 software (Bio‐Rad). Target protein signal was normalized to the total protein signal.

The amounts of Cx43 and Cx43^S368^ in AAC animals were determined using a similar protocol, with rabbit anti‐Cx43 (1:8000, Sigma‐Aldrich #C6219) and rabbit anti‐ Cx43^S368^ (1:5000, Cell Signaling Technology #3511) as primary antibodies. Target protein signal was normalized to a detailed description of the methods is provided in the Supporting Information.

### 4.8 Lipidomics

In the separate sets of AAC animals (Figure 2), rats were killed by cervical dislocation at PD21.

LV was dissected, weighed, and immediately frozen in liquid nitrogen and stored at −80°C until use for lipidomic analyses. Lipids from the LV were extracted according to Folch et al. [123] and lipid classes were separated by thin layer chromatography, fatty acids converted into methyl esters and analyzed by gas chromatography using a gas chromatograph platform consisting of a GC‐FOCUS with an automatic sampler AI 1310 (Thermo Fisher Scientific, Italy). Data underwent log_10_ transformation, centering, and pareto scaling. Fold Change was calculated as the ratio of the raw mean concentration of a lipid metabolite in one group to the raw mean concentration in another group. As biologically significant was considered log_2_FC ≥ 1.2 or log_2_FC ≤ −1.2.

### 4.9 Histology and Immunofluorescence

Hearts collected after optical mapping were fixed in 4% in PBS paraformaldehyde at 4°C for 24 h, embedded in paraplast, and sectioned at 8 μm. Sections were stained with Hematoxylin and Eosin (H&E) or PSR to visualize myocardial fibrosis via collagen I and III [114]. For immunofluorescence, sections were incubated at 4°C overnight with the following selected primary antibodies: rabbit anti‐Cx43 (Sigma, #C6219), rabbit anti‐phospho‐Cx43 Ser368 (Cell Signaling, #3511S), and mouse anti‐N‐cadherin (Thermo Fisher, #33‐3900). Secondary antibodies were applied at room temperature for 90 min. The anti‐N‐cadherin target signal was amplified using immunohistochemical staining with the avidin‐biotin complex (ABC) and labeled extravidin‐biotin (LEAB) methods. Cell boundaries were counterstained with wheat germ agglutinin (WGA) coupled with Alexa 488 (Thermo Fisher, #W11261). Nuclei were counterstained with DAPI (BioVision, # B1098, 1:1000) as described [124].

Transmitted light images were acquired on an Olympus virtual slide scanner (×20 objective), and confocal images were acquired on an Olympus BX61FluoView upright confocal system (Olympus, Japan).

### 4.10 Image Analysis

Quantification of PSR‐positive area, total Cx43, and Cx43^S368^ was performed using FIJI open‐source software. Images were obtained to cover all circular layers of the LV free wall at the level of the papillary muscle (10 ROIs per animal). In AAC animals, Cx43 and Cx43^S368^ localization to ICDs was evaluated by double staining with N‐cadherin (ICD marker). Distance analysis plugin of FIJI was used to assess the proximity of the Cx43 and Cx43^S368^, respectively, with the N‐cadherin. Given the lateral distribution of the N‐cadherin up to PD11 [33], this approach was unfeasible. Therefore, we determined Cx43 distribution by manual selection of the cardiomyocytes and dividing them into the central, intermediate, and end‐located Cx43 groups (Figure 1B).

CSA of cardiomyocytes in papillary muscle and cardiomyocyte width from the circular layer of the LV free wall was determined from WGA‐ and DAPI‐stained sections using Cellpose software (Supporting Information, Figure S2). Cardiomyocyte lengths, width, and cell volume were obtained from the left ventricle free wall circular layer. Cells with clearly visible ICD at both ends were selected for the measurement and analysis of the cell volume (28–44 cells per animal) [60].

### 4.11 Statistical Analysis

Statistical analyses were performed using GraphPad Prism 9 (GraphPad Software, USA). One‐way ANOVA with Tukey multiple comparisons test was used for group comparisons of normally distributed data. Pearson correlation was used to evaluate linear associations between continuous variables.

## 5 Conclusion

Our results demonstrate that electrical remodeling in response to pressure overload is significantly influenced by the timing of the insult relative to the cardiac developmental stage. When LVPO was imposed during the proliferative phase, CV remained preserved, leading to the attenuated pro‐arrhythmogenic environment. These effects were accompanied by a trend toward higher Cx43 and Cx43^S368^ localization at the ICD and stable LCAC levels. In contrast, pressure overload after the proliferative phase resulted in typical conduction slowing and increased LCACs, consistent with maladaptive remodeling seen in adult LVPO. These findings suggest that the developmental state of the myocardium at the time of pressure overload critically determines the electrical phenotype.

## Conflicts of Interest

The authors declare no conflicts of interest.

## Supporting information


Data S1.

## Data Availability

All the data of this study is available from the corresponding author upon a reasonable request.

## References

[1] H. Dai , N. L. Bragazzi , A. Younis , et al., “Worldwide Trends in Prevalence, Mortality, and Disability‐Adjusted Life Years for Hypertensive Heart Disease From 1990 to 2017,” Hypertension 77, no. 4 (2021): 1223–1233. 33583201 10.1161/HYPERTENSIONAHA.120.16483

[2] L. M. Ruilope and R. E. Schmieder , “Left Ventricular Hypertrophy and Clinical Outcomes in Hypertensive Patients,” American Journal of Hypertension 21, no. 5 (2008): 500–508. 18437140 10.1038/ajh.2008.16

[3] C. Cuspidi , C. Sala , F. Negri , G. Mancia , and A. Morganti , “Prevalence of Left‐Ventricular Hypertrophy in Hypertension: An Updated Review of Echocardiographic Studies,” Journal of Human Hypertension 26, no. 6 (2012): 343–349. 22113443 10.1038/jhh.2011.104

[4] W. Joyce , H. A. Shiels , and C. E. Franklin , “The Integrative Biology of the Heart: Mechanisms Enabling Cardiac Plasticity,” Journal of Experimental Biology 227, no. 20 (2024): jeb249348. 39422034 10.1242/jeb.249348

[5] F. Li , X. Wang , J. M. Capasso , and A. M. Gerdes , “Rapid Transition of Cardiac Myocytes From Hyperplasia to Hypertrophy During Postnatal Development,” Journal of Molecular and Cellular Cardiology 28, no. 8 (1996): 1737–1746, 10.1006/jmcc.1996.0163. 8877783

[6] M. Mollova , K. Bersell , S. Walsh , et al., “Cardiomyocyte Proliferation Contributes to Heart Growth in Young Humans,” Proceedings of the National Academy of Sciences of the United States of America 110, no. 4 (2013): 1446–1451. 23302686 10.1073/pnas.1214608110 PMC3557060

[7] T. Aiba and G. F. Tomaselli , “Electrical Remodeling in the Failing Heart,” Current Opinion in Cardiology 25, no. 1 (2010): 29–36. 19907317 10.1097/HCO.0b013e328333d3d6 PMC2855498

[8] Y. Zhao , S. Iyer , M. Tavanaei , N. T. Nguyen , A. Lin , and T. P. Nguyen , “Proarrhythmic Electrical Remodeling by Noncardiomyocytes at Interfaces With Cardiomyocytes Under Oxidative Stress,” Frontiers in Physiology 11 (2021): 622613, 10.3389/fphys.2020.622613. 33603677 PMC7884825

[9] S. B. Danik , G. Rosner , J. Lader , D. E. Gutstein , G. I. Fishman , and G. E. Morley , “Electrical Remodeling Contributes to Complex Tachyarrhythmias in connexin43‐Deficient Mouse Hearts,” FASEB Journal 22, no. 4 (2008): 1204–1212. 17984180 10.1096/fj.07-8974com PMC2726820

[10] D.‐W. Peng , Y.‐Y. Lai , X.‐S. Luo , et al., “Connexin 43 Participates in Atrial Electrical Remodelling Through Colocalization With Calcium Channels in Atrial Myocytes,” Clinical and Experimental Pharmacology & Physiology 49, no. 1 (2022): 25–34. 34438468 10.1111/1440-1681.13580

[11] M. Boulaksil , S. K. G. Winckels , M. A. Engelen , et al., “Heterogeneous Connexin43 Distribution in Heart Failure Is Associated With Dispersed Conduction and Enhanced Susceptibility to Ventricular Arrhythmias,” European Journal of Heart Failure 12, no. 9 (2010): 913–921. 20534605 10.1093/eurjhf/hfq092

[12] A. W. Haider , M. G. Larson , E. J. Benjamin , and D. Levy , “Increased Left Ventricular Mass and Hypertrophy Are Associated With Increased Risk for Sudden Death,” Journal of the American College of Cardiology 32, no. 5 (1998): 1454–1459. 9809962 10.1016/s0735-1097(98)00407-0

[13] D. Levy , K. M. Anderson , D. D. Savage , S. A. Balkus , W. B. Kannel , and W. P. Castelli , “Risk of Ventricular Arrhythmias in Left Ventricular Hypertrophy: The Framingham Heart Study,” American Journal of Cardiology 60, no. 7 (1987): 560–565. 2957907 10.1016/0002-9149(87)90305-5

[14] R. Nadarajah , P. A. Patel , and M. H. Tayebjee , “Is Hypertensive Left Ventricular Hypertrophy a Cause of Sustained Ventricular Arrhythmias in Humans?,” Journal of Human Hypertension 35, no. 6 (2021): 492–498. 33674703 10.1038/s41371-021-00503-w PMC8208890

[15] R. J. Myerburg , “Sudden Cardiac Death: Exploring the Limits of Our Knowledge,” Journal of Cardiovascular Electrophysiology 12, no. 3 (2001): 369–381. 11291815 10.1046/j.1540-8167.2001.00369.x

[16] W. Rosamond , K. Flegal , K. Furie , et al., “Heart Disease and Stroke Statistics—2008 Update: A Report From the American Heart Association Statistics Committee and Stroke Statistics Subcommittee,” Circulation 117, no. 4 (2008): e25–e146. 18086926 10.1161/CIRCULATIONAHA.107.187998

[17] M. Boulaksil , M. F. A. Bierhuizen , M. A. Engelen , et al., “Spatial Heterogeneity of Cx43 Is an Arrhythmogenic Substrate of Polymorphic Ventricular Tachycardias During Compensated Cardiac Hypertrophy in Rats,” Frontiers in Cardiovascular Medicine 3 (2016): 5. 26973841 10.3389/fcvm.2016.00005 PMC4773605

[18] M. S. C. Fontes , A. J. A. Raaijmakers , T. van Doorn , et al., “Changes in Cx43 and NaV1.5 Expression Precede the Occurrence of Substantial Fibrosis in Calcineurin‐Induced Murine Cardiac Hypertrophy,” PLoS One 9, no. 1 (2014): e87226. 24498049 10.1371/journal.pone.0087226 PMC3909068

[19] S. Kostin , S. Dammer , S. Hein , W. P. Klovekorn , E. P. Bauer , and J. Schaper , “Connexin 43 Expression and Distribution in Compensated and Decompensated Cardiac Hypertrophy in Patients With Aortic Stenosis,” Cardiovascular Research 62, no. 2 (2004): 426–436. 15094362 10.1016/j.cardiores.2003.12.010

[20] D. Billur , Y. Olgar , and B. Turan , “Intracellular Redistribution of Left Ventricular Connexin 43 Contributes to the Remodeling of Electrical Properties of the Heart in Insulin‐Resistant Elderly Rats,” Journal of Histochemistry and Cytochemistry 70, no. 6 (2022): 447–462. 35608408 10.1369/00221554221101661 PMC9169104

[21] D. Sedmera , J. Neckar , J. Benes , et al., “Changes in Myocardial Composition and Conduction Properties in Rat Heart Failure Model Induced by Chronic Volume Overload,” Frontiers in Physiology 7 (2016): 367, 10.3389/fphys.2016.00367. 27610087 PMC4997968

[22] P. S. Azevedo , B. F. Polegato , M. F. Minicucci , S. A. R. Paiva , and L. A. M. Zornoff , “Cardiac Remodeling: Concepts, Clinical Impact, Pathophysiological Mechanisms and Pharmacologic Treatment,” Arquivos Brasileiros de Cardiologia 106, no. 1 (2016): 62–69. 26647721 10.5935/abc.20160005 PMC4728597

[23] H. K. Graham , M. Horn , and A. W. Trafford , “Extracellular Matrix Profiles in the Progression to Heart Failure,” Acta Physiologica 194, no. 1 (2008): 3–21. 18577182 10.1111/j.1748-1716.2008.01881.x

[24] H. M. Aitken‐Buck , J. Krause , T. Zeller , P. P. Jones , and R. R. Lamberts , “Long‐Chain Acylcarnitines and Cardiac Excitation‐Contraction Coupling: Links to Arrhythmias,” Frontiers in Physiology 11 (2020): 577856, 10.3389/fphys.2020.577856. 33041874 PMC7518131

[25] M. van Bilsen and A. Planavila , “Fatty Acids and Cardiac Disease: Fuel Carrying a Message,” Acta Physiologica 211, no. 3 (2014): 476–490. 24773697 10.1111/apha.12308

[26] M. Vornanen , “Force‐Frequency Relationship, Contraction Duration and Recirculating Fraction of Calcium in Postnatally Developing Rat Heart Ventricles: Correlation With Heart Rate,” Acta Physiologica Scandinavica 145, no. 4 (1992): 311–321. 1529721 10.1111/j.1748-1716.1992.tb09371.x

[27] S. Kannan and C. Kwon , “Regulation of Cardiomyocyte Maturation During Critical Perinatal Window,” Journal of Physiology 598, no. 14 (2020): 2941–2956. 30571853 10.1113/JP276754 PMC7682257

[28] L. Boulgakoff , R. Sturny , V. Olejnickova , D. Sedmera , R. G. Kelly , and L. Miquerol , “Participation of Ventricular Trabeculae in Neonatal Cardiac Regeneration Leads to Ectopic Recruitment of Purkinje‐Like Cells,” Nature Cardiovascular Research 3, no. 9 (2024): 1140–1157. 10.1038/s44161-024-00530-z 39198628

[29] M. M. Mohammadi , A. Abouissa , I. Azizah , et al., “Induction of Cardiomyocyte Proliferation and Angiogenesis Protects Neonatal Mice From Pressure Overload–Associated Maladaptation,” JCI Insight 5, no. 16 (2019): e128336, 10.1172/jci.insight.128336. 31335322 PMC6777810

[30] B. J. Haubner , M. Adamowicz‐Brice , S. Khadayate , et al., “Complete Cardiac Regeneration in a Mouse Model of Myocardial Infarction,” Aging (Albany NY) 4, no. 12 (2012): 966–977. 23425860 10.18632/aging.100526 PMC3615162

[31] E. R. Porrello , A. I. Mahmoud , E. Simpson , et al., “Transient Regenerative Potential of the Neonatal Mouse Heart,” Science 331, no. 6020 (2011): 1078–1080. 21350179 10.1126/science.1200708 PMC3099478

[32] E. R. Porrello and E. N. Olson , “A Neonatal Blueprint for Cardiac Regeneration,” Stem Cell Research 13, no. 3 Pt B (2014): 556–570. 25108892 10.1016/j.scr.2014.06.003 PMC4316722

[33] B. D. Angst , L. U. Khan , N. J. Severs , et al., “Dissociated Spatial Patterning of Gap Junctions and Cell Adhesion Junctions During Postnatal Differentiation of Ventricular Myocardium,” Circulation Research 80, no. 1 (1997): 88–94. 8978327 10.1161/01.res.80.1.88

[34] A. Vreeker , L. van Stuijvenberg , T. J. Hund , P. J. Mohler , P. G. J. Nikkels , and T. A. B. van Veen , “Assembly of the Cardiac Intercalated Disk During Pre‐ and Postnatal Development of the Human Heart,” PLoS One 9, no. 4 (2014): e94722. 24733085 10.1371/journal.pone.0094722 PMC3986238

[35] J. F. Rhodes , Z. M. Hijazi , and R. J. Sommer , “Pathophysiology of Congenital Heart Disease in the Adult, Part II,” Circulation 117, no. 9 (2008): 1228–1237. 18316499 10.1161/CIRCULATIONAHA.107.742072

[36] K. K. Stout , C. S. Broberg , W. M. Book , et al., “Chronic Heart Failure in Congenital Heart Disease: A Scientific Statement From the American Heart Association,” Circulation 133, no. 8 (2016): 770–801. 26787728 10.1161/CIR.0000000000000352

[37] F. Kolář , F. Papoušek , V. Pelouch , B. Ošťádal , and K. Rakusan , “Pressure Overload Induced in Newborn Rats: Effects on Left Ventricular Growth, Morphology, and Function,” Pediatric Research 43, no. 4 (1998): 521–526. 9545008 10.1203/00006450-199804000-00014

[38] J. Novotny , M. Hrbasová , F. Kolář , and P. Svoboda , “Cardiomegaly Induced by Pressure Overload in Newborn Rats Is Accompanied by Altered Expression of the Long Isoform of Gsα Protein and Deranged Signaling of Adenylyl Cyclase,” Molecular and Cellular Biochemistry 245, no. 1 (2003): 157–166. 12708755 10.1023/a:1022828430565

[39] D. Sedmera , R. P. Thompson , and F. Kolar , “Effect of Increased Pressure Loading on Heart Growth in Neonatal Rats,” Journal of Molecular and Cellular Cardiology 35, no. 3 (2003): 301–309. 12676545 10.1016/s0022-2828(03)00011-7

[40] R. E. Ahmed , T. Tokuyama , T. Anzai , N. Chanthra , and H. Uosaki , “Sarcomere Maturation: Function Acquisition, Molecular Mechanism, and Interplay With Other Organelles,” Philosophical Transactions of the Royal Society, B: Biological Sciences 377 (2022): 20210325, 10.1098/rstb.2021.0325. PMC9527934 36189811

[41] S. E. Campbell , K. Rakusan , and A. M. Gerdes , “Change in Cardiac Myocyte Size Distribution in Aortic‐Constricted Neonatal Rats,” Basic Research in Cardiology 84, no. 3 (1989): 247–258. 10.1007/BF01907972 2764857

[42] M. M. J. Nassal , A. A. Werdich , X. Wan , et al., “Phosphorylation at Connexin43 Serine‐368 Is Necessary for Myocardial Conduction During Metabolic Stress,” Journal of Cardiovascular Electrophysiology 27, no. 1 (2016): 110–119. 26459193 10.1111/jce.12833 PMC4879887

[43] M. Dambrova , M. Makrecka‐Kuka , J. Kuka , et al., “Acylcarnitines: Nomenclature, Biomarkers, Therapeutic Potential, Drug Targets, and Clinical Trials,” Pharmacological Reviews 74, no. 3 (2022): 506–551. 35710135 10.1124/pharmrev.121.000408

[44] J. H. Rennison and D. R. Van Wagoner , “Impact of Dietary Fatty Acids on Cardiac Arrhythmogenesis,” Circulation: Arrhythmia and Electrophysiology 2, no. 4 (2009): 460–469. 19808503 10.1161/CIRCEP.109.880773 PMC5831114

[45] J. Krause , A. Nickel , A. Madsen , et al., “An Arrhythmogenic Metabolite in Atrial Fibrillation,” Journal of Translational Medicine 21 (2023): 566. 37620858 10.1186/s12967-023-04420-z PMC10464005

[46] X. Ai and S. M. Pogwizd , “Connexin 43 Downregulation and Dephosphorylation in Nonischemic Heart Failure Is Associated With Enhanced Colocalized Protein Phosphatase Type 2A,” Circulation Research 96, no. 1 (2005): 54–63. 15576650 10.1161/01.RES.0000152325.07495.5a

[47] J. Yan , C. Killingsworth , G. Walcott , et al., “Molecular Remodeling of Cx43, but Not Structural Remodeling, Promotes Arrhythmias in an Arrhythmogenic Canine Model of Nonischemic Heart Failure,” Journal of Molecular and Cellular Cardiology 158 (2021): 72–81. 34048725 10.1016/j.yjmcc.2021.05.012 PMC8963384

[48] S. Salameh , V. Ogueri , and N. G. Posnack , “Adapting to a New Environment: Postnatal Maturation of the Human Cardiomyocyte,” Journal of Physiology 601, no. 13 (2023): 2593–2619. 37031380 10.1113/JP283792 PMC10775138

[49] J. Zhang , L. Cao , Y. Tan , Y. Zheng , and Y. Gui , “ *N*‐Acetylcysteine Protects Neonatal Mice From Ventricular Hypertrophy Induced by Maternal Obesity in a Sex‐Specific Manner,” Biomedicine & Pharmacotherapy 133 (2021): 110989. 33378994 10.1016/j.biopha.2020.110989

[50] Y. Li , J. Ke , C. Peng , F. Wu , and Y. Song , “microRNA‐300/NAMPT Regulates Inflammatory Responses Through Activation of AMPK/mTOR Signaling Pathway in Neonatal Sepsis,” Biomedicine & Pharmacotherapy 108 (2018): 271–279. 30223098 10.1016/j.biopha.2018.08.064

[51] B. J. Haubner , J. Schneider , U. Schweigmann , et al., “Functional Recovery of a Human Neonatal Heart After Severe Myocardial Infarction,” Circulation Research 118, no. 2 (2016): 216–221. 26659640 10.1161/CIRCRESAHA.115.307017

[52] X. Ding , S. Wang , Y. Wang , et al., “Neonatal Heart Responds to Pressure Overload With Differential Alterations in Various Cardiomyocyte Maturation Programs That Accommodate Simultaneous Hypertrophy and Hyperplasia,” Frontiers in Cell and Development Biology 8 (2020): 596960. 10.3389/fcell.2020.596960 PMC7710899 33330485

[53] I. Jarabicová , C. Horváth , J. Hrdlička , et al., “Necrosis‐Like Cell Death Modes in Heart Failure: The Influence of Aetiology and the Effects of RIP3 Inhibition,” Basic Research in Cardiology 120, no. 2 (2025): 373–392. 40088261 10.1007/s00395-025-01101-4 PMC11976840

[54] X. Koenig , S. Dysek , S. Kimbacher , et al., “Voltage‐Gated Ion Channel Dysfunction Precedes Cardiomyopathy Development in the Dystrophic Heart,” PLoS One 6, no. 5 (2011): e20300. 21677768 10.1371/journal.pone.0020300 PMC3100353

[55] X. Koenig , L. Rubi , G. J. Obermair , et al., “Enhanced Currents Through L‐Type Calcium Channels in Cardiomyocytes Disturb the Electrophysiology of the Dystrophic Heart,” American Journal of Physiology ‐ Heart and Circulatory Physiology 306, no. 4 (2014): H564–H573. 24337461 10.1152/ajpheart.00441.2013 PMC4892346

[56] L. Rubi , V. S. Gawali , H. Kubista , H. Todt , K. Hilber , and X. Koenig , “Proper Voltage‐Dependent Ion Channel Function in Dysferlin‐Deficient Cardiomyocytes,” Cellular Physiology and Biochemistry 36, no. 3 (2015): 1049–1058. 26112643 10.1159/000430278

[57] L. Rubi , H. Todt , H. Kubista , X. Koenig , and K. Hilber , “Calcium Current Properties in Dystrophin‐Deficient Ventricular Cardiomyocytes From Aged mdx Mice,” Physiological Reports 6, no. 1 (2018): e13567. 29333726 10.14814/phy2.13567 PMC5789658

[58] M. Mirza , A. Strunets , W.‐K. Shen , and A. Jahangir , “Mechanisms of Arrhythmias and Conduction Disorders in Older Adults,” Clinics in Geriatric Medicine 28, no. 4 (2012): 555–573. 23101571 10.1016/j.cger.2012.08.005 PMC3610528

[59] D. Wulfsohn , J. R. Nyengaard , and Y. Tang , “Postnatal Growth of Cardiomyocytes in the Left Ventricle of the Rat,” Anatomical Record. Part A, Discoveries in Molecular, Cellular, and Evolutionary Biology 277, no. 1 (2004): 236–247. 14983518 10.1002/ar.a.20009

[60] B. Korecky and K. Rakusan , “Normal and Hypertrophic Growth of the Rat Heart: Changes in Cell Dimensions and Number,” American Journal of Physiology 234, no. 2 (1978): H123–H128. 146437 10.1152/ajpheart.1978.234.2.H123

[61] P. Anversa , G. Olivetti , and A. V. Loud , “Morphometric Study of Early Postnatal Development in the Left and Right Ventricular Myocardium of the Rat. I. Hypertrophy, Hyperplasia, and Binucleation of Myocytes,” Circulation Research 46, no. 4 (1980): 495–502. 6444554 10.1161/01.res.46.4.495

[62] T. M. Mayhew , A. Pharaoh , A. Austin , and D. G. Fagan , “Stereological Estimates of Nuclear Number in Human Ventricular Cardiomyocytes Before and After Birth Obtained Using Physical Disectors,” Journal of Anatomy 191, no. Pt 1 (1997): 107–115. 9279664 10.1046/j.1469-7580.1997.19110107.x PMC1467664

[63] K. T. Haq , K. McLean , S. Salameh , L. M. Swift , and N. G. Posnack , “Electroanatomical Adaptations in the Guinea Pig Heart From Neonatal to Adulthood,” Europace: European Pacing, Arrhythmias, and Cardiac Electrophysiology: Journal of the Working Groups on Cardiac Pacing, Arrhythmias, and Cardiac Cellular Electrophysiology of the European Society of Cardiology 26, no. 7 (2024): euae158. 38864516 10.1093/europace/euae158 PMC11218563

[64] M. S. Spach , J. F. Heidlage , P. C. Dolber , and R. C. Barr , “Electrophysiological Effects of Remodeling Cardiac Gap Junctions and Cell Size,” Circulation Research 86, no. 3 (2000): 302–311. 10679482 10.1161/01.res.86.3.302

[65] K. T. Haq , B. L. Cooper , F. Berk , and N. G. Posnack , “The Effect of Sex and Age on Ex Vivo Cardiac Electrophysiology: Insight From a Guinea Pig Model,” American Journal of Physiology ‐ Heart and Circulatory Physiology 324, no. 1 (2023): H141–H154. 36487188 10.1152/ajpheart.00497.2022 PMC9829463

[66] I. M. Ruhr , H. A. Shiels , D. A. Crossley , and G. L. J. Galli , “Developmental Programming of Sarcoplasmic Reticulum Function Improves Cardiac Anoxia Tolerance in Turtles,” Journal of Experimental Biology 227, no. 20 (2024): jeb247434. 39246147 10.1242/jeb.247434

[67] L. M. Swift , M. Burke , D. Guerrelli , et al., “Age‐Dependent Changes in Electrophysiology and Calcium Handling: Implications for Pediatric Cardiac Research,” American Journal of Physiology. Heart and Circulatory Physiology 318, no. 2 (2020): H354–H365. 31886723 10.1152/ajpheart.00521.2019 PMC7052624

[68] F. J. Clubb and S. P. Bishop , “Formation of Binucleated Myocardial Cells in the Neonatal Rat. An Index for Growth Hypertrophy,” Laboratory Investigation 50, no. 5 (1984): 571–577. 6232423

[69] C. de Diego , F. Chen , Y. Xie , et al., “Anisotropic Conduction Block and Reentry in Neonatal Rat Ventricular Myocyte Monolayers,” American Journal of Physiology. Heart and Circulatory Physiology 300, no. 1 (2011): H271–H278. 21037233 10.1152/ajpheart.00758.2009 PMC3023258

[70] B. Burstein and S. Nattel , “Atrial Fibrosis: Mechanisms and Clinical Relevance in Atrial Fibrillation,” Journal of the American College of Cardiology 51, no. 8 (2008): 802–809. 18294563 10.1016/j.jacc.2007.09.064

[71] H. Kitamura , Y. Ohnishi , A. Yoshida , et al., “Heterogeneous Loss of connexin43 Protein in Nonischemic Dilated Cardiomyopathy With Ventricular Tachycardia,” Journal of Cardiovascular Electrophysiology 13, no. 9 (2002): 865–870. 12380923 10.1046/j.1540-8167.2002.00865.x

[72] H. Kitamura , A. Yoshida , Y. Ohnishi , et al., “Correlation of connexin43 Expression and Late Ventricular Potentials in Nonischemic Dilated Cardiomyopathy,” Circulation Journal 67, no. 12 (2003): 1017–1021. 14639017 10.1253/circj.67.1017

[73] A. Salameh , S. Krautblatter , S. Karl , et al., “The Signal Transduction Cascade Regulating the Expression of the Gap Junction Protein connexin43 by Beta‐Adrenoceptors,” British Journal of Pharmacology 158, no. 1 (2009): 198–208. 19719782 10.1111/j.1476-5381.2009.00344.x PMC2795252

[74] M. Uzzaman , H. Honjo , Y. Takagishi , et al., “Remodeling of Gap Junctional Coupling in Hypertrophied Right Ventricles of Rats With Monocrotaline‐Induced Pulmonary Hypertension,” Circulation Research 86, no. 8 (2000): 871–878. 10785509 10.1161/01.res.86.8.871

[75] H. Takanari , V. J. A. Bourgonje , M. S. C. Fontes , et al., “Calmodulin/CaMKII Inhibition Improves Intercellular Communication and Impulse Propagation in the Heart and Is Antiarrhythmic Under Conditions When Fibrosis Is Absent,” Cardiovascular Research 111, no. 4 (2016): 410–421. 27357638 10.1093/cvr/cvw173 PMC4996261

[76] P. S. Dhillon , R. Gray , P. Kojodjojo , et al., “Relationship Between Gap‐Junctional Conductance and Conduction Velocity in Mammalian Myocardium,” Circulation. Arrhythmia and Electrophysiology 6, no. 6 (2013): 1208–1214. 24134868 10.1161/CIRCEP.113.000848

[77] M. S. Spach , P. C. Dolber , and J. F. Heidlage , “Influence of the Passive Anisotropic Properties on Directional Differences in Propagation Following Modification of the Sodium Conductance in Human Atrial Muscle. A Model of Reentry Based on Anisotropic Discontinuous Propagation,” Circulation Research 62, no. 4 (1988): 811–832. 2450697 10.1161/01.res.62.4.811

[78] S. P. Thomas , J. P. Kucera , L. Bircher‐Lehmann , Y. Rudy , J. E. Saffitz , and A. G. Kléber , “Impulse Propagation in Synthetic Strands of Neonatal Cardiac Myocytes With Genetically Reduced Levels of connexin43,” Circulation Research 92, no. 11 (2003): 1209–1216. 12730095 10.1161/01.RES.0000074916.41221.EA PMC2242733

[79] S. Ozaki , H. Nakaya , Y. Gotoh , M. Azuma , O. Kemmotsu , and M. Kanno , “Effects of Halothane and Enflurane on Conduction Velocity and Maximum Rate of Rise of Action Potential Upstroke in Guinea Pig Papillary Muscles,” Anesthesia and Analgesia 68, no. 3 (1989): 219–225. 2919756

[80] S. Rohr , J. P. Kucera , and A. G. Kléber , “Slow Conduction in Cardiac Tissue, I: Effects of a Reduction of Excitability Versus a Reduction of Electrical Coupling on Microconduction,” Circulation Research 83, no. 8 (1998): 781–794. 9776725 10.1161/01.res.83.8.781

[81] M. Entz , S. A. George , M. J. Zeitz , T. Raisch , J. W. Smyth , and S. Poelzing , “Heart Rate and Extracellular Sodium and Potassium Modulation of Gap Junction Mediated Conduction in Guinea Pigs,” Frontiers in Physiology 7 (2016): 16. 26869934 10.3389/fphys.2016.00016 PMC4735342

[82] L. Bosch , J. J. de Haan , M. Bastemeijer , et al., “The Transverse Aortic Constriction Heart Failure Animal Model: A Systematic Review and Meta‐Analysis,” Heart Failure Reviews 26, no. 6 (2021): 1515–1524. 32335789 10.1007/s10741-020-09960-w PMC8510918

[83] M. Sykora , V. Kratky , L. Kopkan , N. Tribulova , and B. Szeiffova Bacova , “Anti‐Fibrotic Potential of Angiotensin (1‐7) in Hemodynamically Overloaded Rat Heart,” International Journal of Molecular Sciences 24, no. 4 (2023): 3490. 36834901 10.3390/ijms24043490 PMC9967643

[84] J. S. Axelsen , S. Andersen , S. Ringgaard , et al., “Right Ventricular Diastolic Adaptation to Pressure Overload in Different Rat Strains,” Physiological Reports 12, no. 13 (2024): e16132. 38993022 10.14814/phy2.16132 PMC11239975

[85] S. Tannu , J. Allocco , M. Yarde , P. Wong , and X. Ma , “Experimental Model of Congestive Heart Failure Induced by Transverse Aortic Constriction in BALB/c Mice,” Journal of Pharmacological and Toxicological Methods 106 (2020): 106935. 33096237 10.1016/j.vascn.2020.106935

[86] M. Zi , N. Stafford , S. Prehar , et al., “Cardiac Hypertrophy or Failure? ‐ A Systematic Evaluation of the Transverse Aortic Constriction Model in C57BL/6NTac and C57BL/6J Substrains,” Current Research in Physiology 1 (2019): 1–10. 32699840 10.1016/j.crphys.2019.10.001 PMC7357793

[87] F. Novák , F. Kolář , S. Voců , M. Vecka , and O. Nováková , “Pressure Overload Selectively Increases n‐3 PUFA in Myocardial Phospholipids During Early Postnatal Period,” Physiological Research 61, no. Suppl 1 (2012): S155–S163. 22827872 10.33549/physiolres.932401

[88] J. Salatzki , A. Foryst‐Ludwig , K. Bentele , et al., “Adipose Tissue ATGL Modifies the Cardiac Lipidome in Pressure‐Overload‐Induced Left Ventricular Failure,” PLoS Genetics 14, no. 1 (2018): e1007171. 29320510 10.1371/journal.pgen.1007171 PMC5779697

[89] J. D. Joss , J. Hernan , R. Collier , and A. Cardenas , “Perioperative Supplementation of Polyunsaturated Omega‐3 Fatty Acid for the Prevention of Atrial Fibrillation After Cardiothoracic Surgery,” American Journal of Health‐System Pharmacy: AJHP: Official Journal of the American Society of Health‐System Pharmacists 74, no. 1 (2017): e17–e23, 10.2146/ajhp150740. 28007717

[90] X. Jia , F. Gao , J. K. Pickett , et al., “Association Between Omega‐3 Fatty Acid Treatment and Atrial Fibrillation in Cardiovascular Outcome Trials: A Systematic Review and Meta‐Analysis,” Cardiovascular Drugs and Therapy 35, no. 4 (2021): 793–800, 10.1007/s10557-021-07204-z. 34057665

[91] I. García‐Lunar , I. Jorge , J. Sáiz , et al., “Metabolic Changes Contribute to Maladaptive Right Ventricular Hypertrophy in Pulmonary Hypertension Beyond Pressure Overload: An Integrative Imaging and Omics Investigation,” Basic Research in Cardiology 119, no. 3 (2024): 419–433. 38536505 10.1007/s00395-024-01041-5 PMC11143050

[92] R. Ji , H. Akashi , K. Drosatos , et al., “Increased De Novo Ceramide Synthesis and Accumulation in Failing Myocardium,” JCI Insight 2, no. 9 (2017): e82922, 10.1172/jci.insight.82922. 28469091 PMC5414571

[93] L. B. Bockus , P. N. Jensen , A. M. Fretts , et al., “Plasma Ceramides and Sphingomyelins and Sudden Cardiac Death in the Cardiovascular Health Study,” JAMA Network Open 6, no. 11 (2023): e2343854. 37976059 10.1001/jamanetworkopen.2023.43854 PMC10656644

[94] D. B. O. van , M. Schuldt , S. Algül , et al., “Metabolomics in Severe Aortic Stenosis Reveals Intermediates of Nitric Oxide Synthesis as Most Distinctive Markers,” International Journal of Molecular Sciences 22, no. 7 (2021): 3569. 33808189 10.3390/ijms22073569 PMC8037707

[95] M. K. Patel , A. P. Economides , and N. G. Byrne , “Effects of Palmitoyl Carnitine on Perfused Heart and Papillary Muscle,” Journal of Cardiovascular Pharmacology and Therapeutics 4, no. 2 (1999): 85–96. 10684527 10.1177/107424849900400203

[96] S. D. DaTorre , M. H. Creer , S. M. Pogwizd , and P. B. Corr , “Amphipathic Lipid Metabolites and Their Relation to Arrhythmogenesis in the Ischemic Heart,” Journal of Molecular and Cellular Cardiology 23, no. Suppl 1 (1991): 11–22. 2038071 10.1016/0022-2828(91)90019-i

[97] S. Joukar , “A Comparative Review on Heart Ion Channels, Action Potentials and Electrocardiogram in Rodents and Human: Extrapolation of Experimental Insights to Clinic,” Laboratory Animal Research 37, no. 1 (2021): 25. 34496976 10.1186/s42826-021-00102-3 PMC8424989

[98] A. G. Edwards and W. E. Louch , “Species‐Dependent Mechanisms of Cardiac Arrhythmia: A Cellular Focus,” Clinical Medicine Insights. Cardiology 11 (2017): 1179546816686061. 28469490 10.1177/1179546816686061 PMC5392019

[99] W. B. van Ham , E. E. M. Meijboom , M. L. Ligtermoet , P. G. J. Nikkels , and T. A. B. van Veen , “Maturation and Function of the Intercalated Disc: Report of Two Pediatric Cases Focusing on Cardiac Development and Myocardial Hyperplasia,” Journal of Cardiovascular Development and Disease 10, no. 8 (2023): 354. 37623366 10.3390/jcdd10080354 PMC10455643

[100] M. J. van Kempen , I. ten Velde , A. Wessels , et al., “Differential Connexin Distribution Accommodates Cardiac Function in Different Species,” Microscopy Research and Technique 31, no. 5 (1995): 420–436. 8534903 10.1002/jemt.1070310511

[101] M. J. A. Van Kempen , J. L. M. Vermeulen , A. F. M. Moorman , D. Gros , D. L. Paul , and W. H. Lamers , “Developmental Changes of connexin40 and connexin43 mRNA Distribution Patterns in the Rat Heart,” Cardiovascular Research 32, no. 5 (1996): 886–900. 8944820 10.1016/0008-6363(96)00131-9

[102] H. S. Chkourko , G. Guerrero‐Serna , X. Lin , et al., “Remodeling of Mechanical Junctions and of Microtubule‐Associated Proteins Accompany Cardiac connexin43 Lateralization,” Heart Rhythm 9, no. 7 (2012): 1133–1140. 22406144 10.1016/j.hrthm.2012.03.003 PMC3723688

[103] M. Günthel , P. Barnett , and V. M. Christoffels , “Development, Proliferation, and Growth of the Mammalian Heart,” Molecular Therapy 26, no. 7 (2018): 1599–1609. 29929790 10.1016/j.ymthe.2018.05.022 PMC6037201

[104] D. Sedmera and R. P. Thompson , “Myocyte Proliferation in the Developing Heart,” Developmental Dynamics 240, no. 6 (2011): 1322–1334. 21538685 10.1002/dvdy.22650 PMC3271704

[105] L. Nt and S. Ha , “Neonatal Heart Regeneration: Comprehensive Literature Review,” Circulation 138, no. 4 (2018): 412–423, 10.1161/CIRCULATIONAHA.118.033648. 30571359 PMC6673675

[106] W. Zhu , E. Zhang , M. Zhao , et al., “Regenerative Potential of Neonatal Porcine Hearts,” Circulation 138, no. 24 (2018): 2809–2816. 30030418 10.1161/CIRCULATIONAHA.118.034886 PMC6301098

[107] L. Ye , G. D'Agostino , S. J. Loo , et al., “Early Regenerative Capacity in the Porcine Heart,” Circulation 138, no. 24 (2018): 2798–2808. 30030417 10.1161/CIRCULATIONAHA.117.031542

[108] S. Fratz , A. Hager , C. Schreiber , M. Schwaiger , J. Hess , and H. C. Stern , “Long‐Term Myocardial Scarring After Operation for Anomalous Left Coronary Artery From the Pulmonary Artery,” Annals of Thoracic Surgery 92, no. 5 (2011): 1761–1765. 22051271 10.1016/j.athoracsur.2011.06.021

[109] J. Neckář , P. Alánová , V. Olejníčková , et al., “Excess Ischemic Tachyarrhythmias Trigger Protection Against Myocardial Infarction in Hypertensive Rats,” Clinical Science (London, England) 135, no. 17 (2021): 2143–2163. 10.1042/CS20210648 34486670

[110] B. Hackl , E. Zabrodska , S. Gewessler , et al., “The Type of Suture Material Affects Transverse Aortic Constriction‐Induced Heart Failure Development in Mice: A Repeated Measures Correlation Analysis,” Frontiers in Cardiovascular Medicine 10 (2023): 1242763. 37795481 10.3389/fcvm.2023.1242763 PMC10546326

[111] J. Kolarova , M. Novakova , M. Ronzhina , et al., “Isolated Rabbit Hearts‐Databases of EGs and MAP Signals,” in Computing in Cardiology Conference (CinC) (IEEE, 2013), 551–554.

[112] H. Tolboom , V. Olejníčková , D. Reser , et al., “Moderate Hypothermia During Ex Vivo Machine Perfusion Promotes Recovery of Hearts Donated After Cardiocirculatory Death†,” European Journal of Cardio‐Thoracic Surgery 49, no. 1 (2016): 25–31. 25740820 10.1093/ejcts/ezv066

[113] V. Olejnickova , P. U. Hamor , J. Janacek , et al., “Development of Ventricular Trabeculae Affects Electrical Conduction in the Early Endothermic Heart,” Developmental Dynamics 253, no. 1 (2024): 78–90. 36400745 10.1002/dvdy.552

[114] E. Zabrodska , A. Kvasilova , D. Sedmera , and V. Olejnickova , “Electrical Remodeling of Atrioventricular Junction: A Study on Retrogradely Perfused Chick Embryonic Heart,” American Journal of Physiology. Heart and Circulatory Physiology 327, no. 3 (2024): H555–H564. 39028286 10.1152/ajpheart.00115.2024 PMC11427115

[115] V. Olejnickova and D. Sedmera , “What Is the Optimal Light Source for Optical Mapping Using Voltage‐ and Calcium‐Sensitive Dyes?,” Physiological Research 69, no. 4 (2020): 599–607. 32584139 10.33549/physiolres.934471 PMC8549889

[116] L. M. Swift , H. Asfour , N. G. Posnack , A. Arutunyan , M. W. Kay , and N. Sarvazyan , “Properties of Blebbistatin for Cardiac Optical Mapping and Other Imaging Applications,” Pflugers Archiv: European Journal of Physiology 464, no. 5 (2012): 503–512. 22990759 10.1007/s00424-012-1147-2 PMC3586237

[117] G. E. Morley , D. Vaidya , F. H. Samie , C. Lo , M. Delmar , and J. Jalife , “Characterization of Conduction in the Ventricles of Normal and Heterozygous Cx43 Knockout Mice Using Optical Mapping,” Journal of Cardiovascular Electrophysiology 10, no. 10 (1999): 1361–1375. 10515561 10.1111/j.1540-8167.1999.tb00192.x

[118] V. Olejnickova , H. Kolesova , M. Bartos , D. Sedmera , and M. Gregorovicova , “The Tale‐Tell Heart: Evolutionary Tetrapod Shift From Aquatic to Terrestrial Life‐Style Reflected in Heart Changes in Axolotl ( *Ambystoma mexicanum* ),” Developmental Dynamics 251, no. 6 (2022): 1004–1014. 34423892 10.1002/dvdy.413

[119] A. Kvasilova , V. Olejnickova , B. Jensen , et al., “The Formation of the Atrioventricular Conduction Axis Is Linked in Development to Ventricular Septation,” Journal of Experimental Biology 223, no. Pt 19 (2020): jeb229278, 10.1242/jeb.229278. 33046580

[120] A. A. Kondratyev , J. G. C. Ponard , A. Munteanu , S. Rohr , and J. P. Kucera , “Dynamic Changes of Cardiac Conduction During Rapid Pacing,” American Journal of Physiology. Heart and Circulatory Physiology 292, no. 4 (2007): H1796–H1811. 17142344 10.1152/ajpheart.00784.2006

[121] C. O'Shea , A. P. Holmes , J. Winter , et al., “Cardiac Optogenetics and Optical Mapping – Overcoming Spectral Congestion in all‐Optical Cardiac Electrophysiology,” Frontiers in Physiology 10 (2019): 182, 10.3389/fphys.2019.00182. 30899227 PMC6416196

[122] A. F. Bruce , S. Rothery , E. Dupont , and N. J. Severs , “Gap Junction Remodelling in Human Heart Failure Is Associated With Increased Interaction of connexin43 With ZO‐1,” Cardiovascular Research 77, no. 4 (2008): 757–765. 18056766 10.1093/cvr/cvm083 PMC5436744

[123] J. Folch , M. Lees , and G. H. S. Stanley , “A Simple Method for the Isolation and Purification of Total Lipides From Animal Tissues,” Journal of Biological Chemistry 226, no. 1 (1957): 497–509. 13428781

[124] V. Olejnickova , M. Kocka , A. Kvasilova , et al., “Gap Junctional Communication via Connexin43 Between Purkinje Fibers and Working Myocytes Explains the Epicardial Activation Pattern in the Postnatal Mouse Left Ventricle,” International Journal of Molecular Sciences 22, no. 5 (2021): 2475. 33804428 10.3390/ijms22052475 PMC7957598

