# Supplementary material for: Electrical Remodeling of Pressure Overloaded Rat Heart Is Attenuated if Imposed During Proliferative Cardiac Growth
Source: Acta Physiol (Oxf). 2025 Oct 15;241(11):e70118. doi: 10.1111/apha.70118 (PMC12522080; doi:10.1111/apha.70118)
Supplement: Supplementary file 1 — Data S1. [file APHA-241-e70118-s001.docx]

**Supplementary Figures**

Figure S1


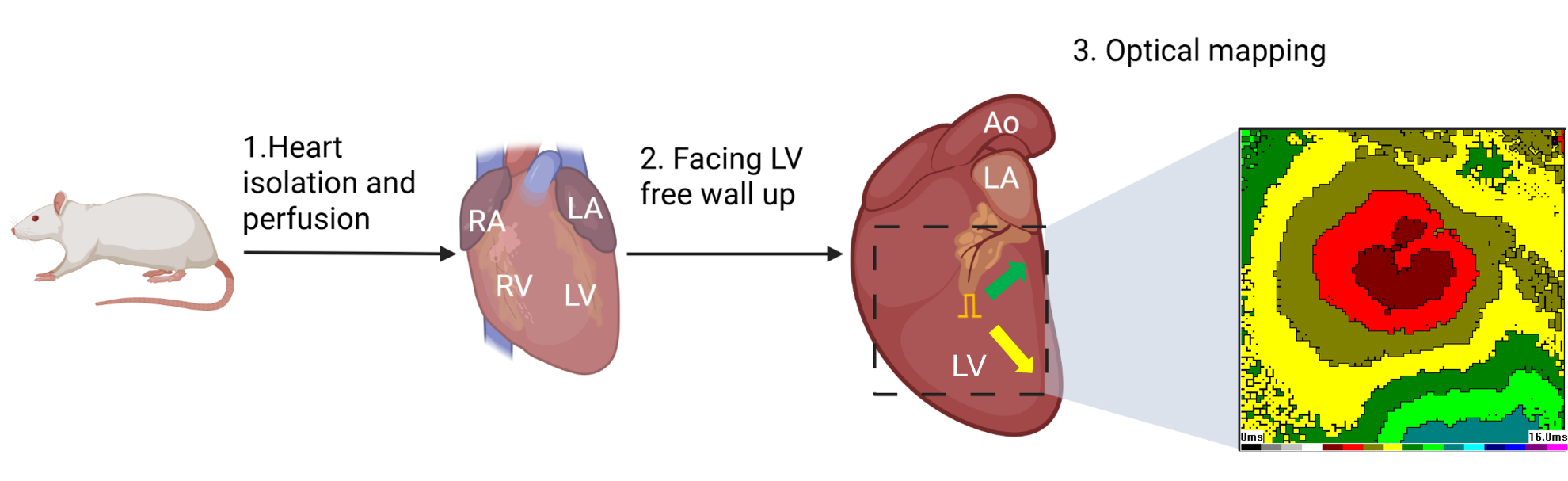


**Figure S1. Experimental approach for optical mapping**

Hearts isolation was followed by Langenforff perfusion (1) and heart placement under the optical mapping setup to image the left ventricle free (THT Mesoscope - Brain Vision Inc., Japan and ULTIMA L camera - SciMedia, Japan) (2). The optical map was obtained under the stimulated rhythm with pacing from the center of the left ventricular free wall and conduction velocity analyzed at the longitudinal (CV_L_, yellow arrow) and transversal (CV_T_, green arrow) direction (3).

Ao, aorta; LA, left atrium; LV, left ventricle; RA, right atrium; RV, right ventricle

Figure was created using Biorender.com

Figure S2


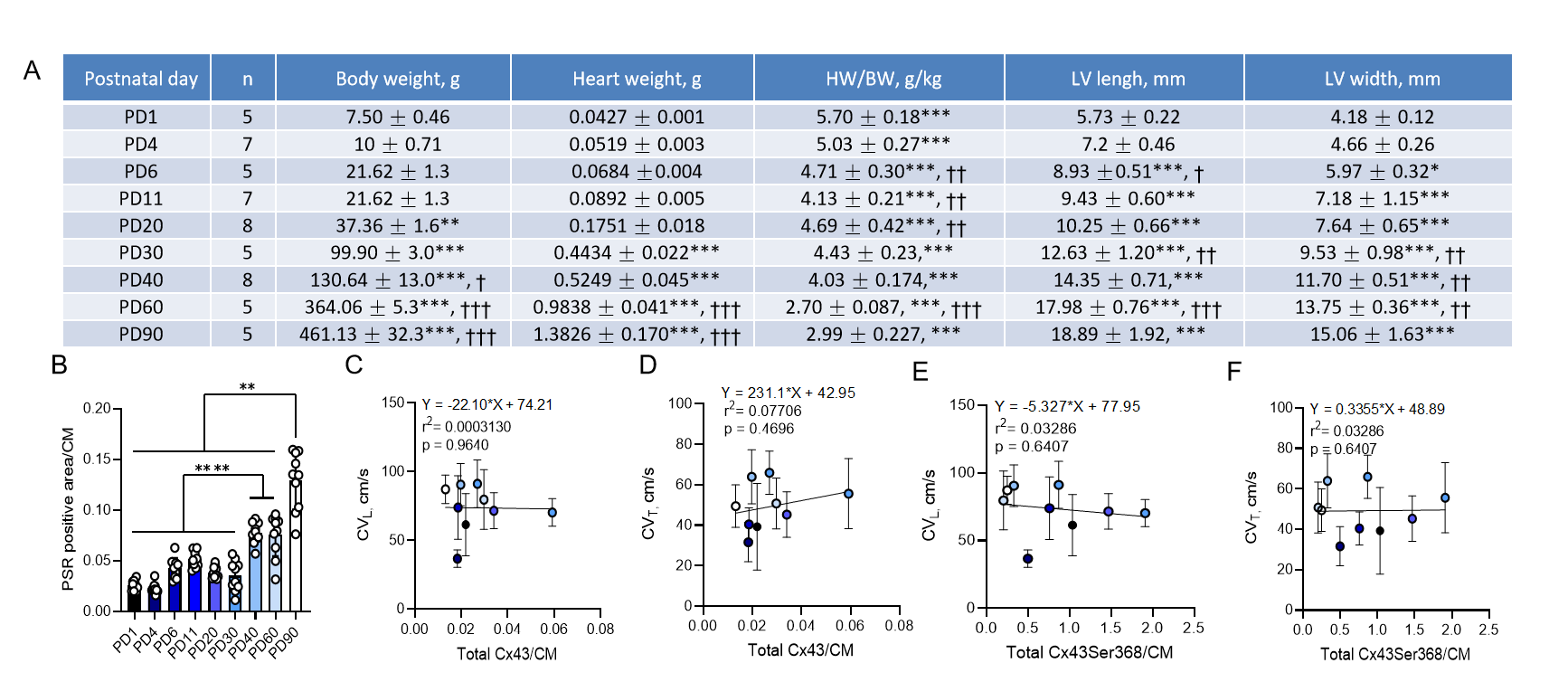


**Figure S2. Morphological parameters, myocardial fibrosis, immunohistochemically detected Cx43 and Cx43^S368^ throughout postnatal rat heart development**

Summary of the changes in morphological parameters during postnatal growth (A). Quantification of the fibrosis using Picrosirius Red (PSR) (B). Correlation of the CV_L_ (C) and CV_T_ (D) with Cx43 normalized to cardiomyocyte. Correlation of the CV_L_ (E) and CV_T_ (F) with Cx43^S368^ normalized to cardiomyocyte.

Data are expressed as mean ± D; one-way ANOVA with Tukey multiple comparisons test was used for A–B; *p < 0.05, **p < 0.01, ***p < 0.001. †p < 0.05, ††p < 0.01, †††p < 0.001 vs. previous developmental stage. Pearson correlation coefficient for C-F.

Figure S3


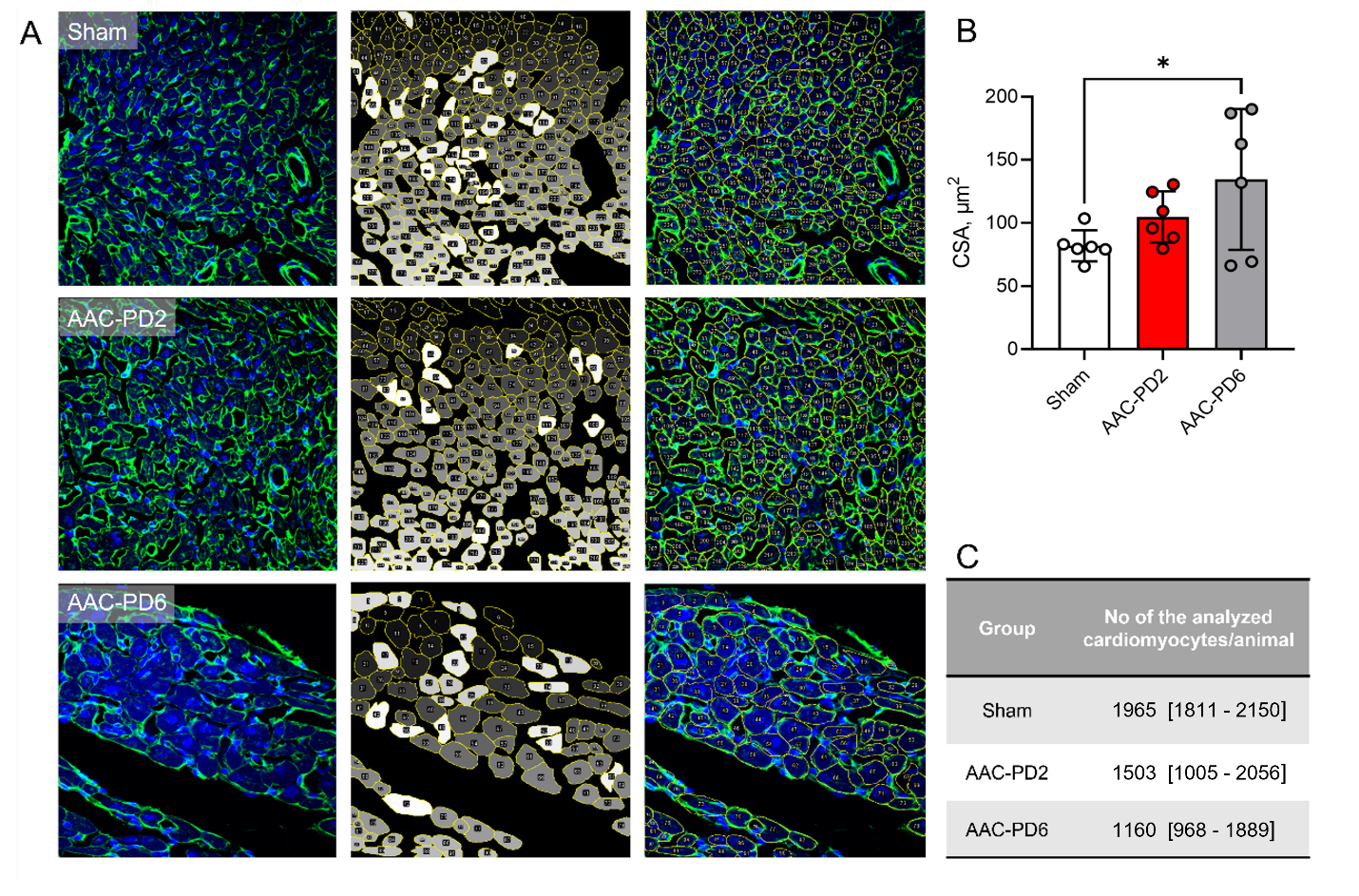


**Figure S3. Stable cardiomyocyte cross-section area in AAC-PD2 hearts**

Representative image illustrating the quantification of cardiomyocyte cross-section area (CSA) from the papillary muscle (A). Quantification of the CSA in experimental groups (B; 6 animals per group, 5 section per animal). Average number of the measured cardiomyocytes per animal with range in the brackets (C).

Data are expressed as mean ± SD; one-way ANOVA with Tukey multiple comparisons test, * p < 0.05.

Figure S4

**
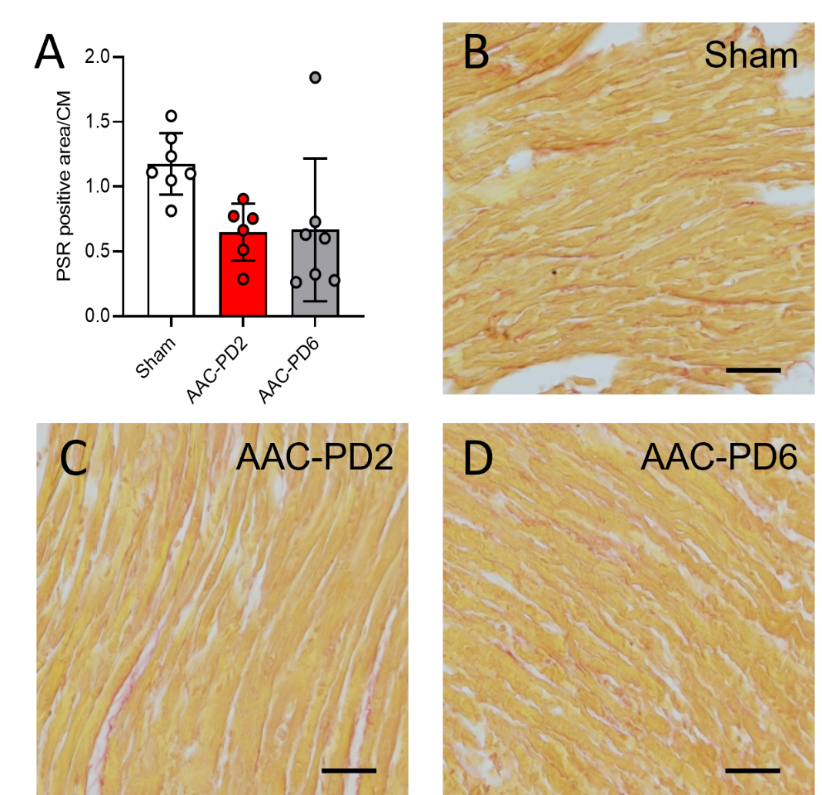
**

**Figure S4. Fibrosis of the AAC-PD2 hearts**

Quantification of myocardial fibrosis detected by Picrosirius Red staining (A). Representative images of the Sham (B), AAC-PD (C), and AAC-PD6 (D) hearts.

Data are expressed as mean ± SD; one-way ANOVA with Tukey multiple comparisons test,

Scale bar represent 50 µm.

Figure S5


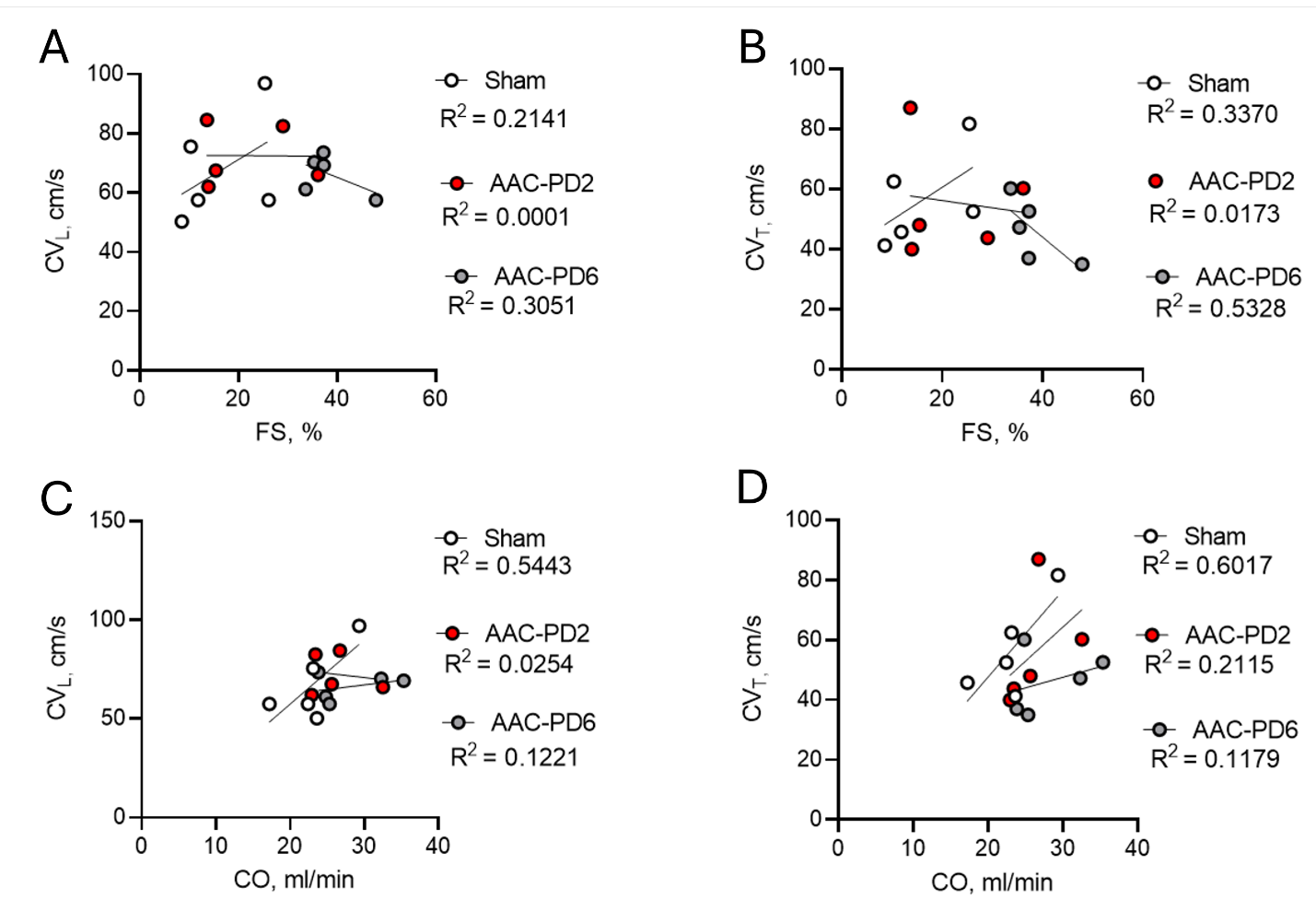


**Figure S5. Electrical characteristics do not correlate with functional parameters in AAC animals**

Correlation between fractional shortening (FS) and longitudinal conduction velocity - CV_L_ (A) and transversal conduction velocity - CV_T_ (B). Correlation between cardiac output (CO) and CV_L_ (C) and CV_T_ (D). Pearson correlation coefficient.

Figure S6


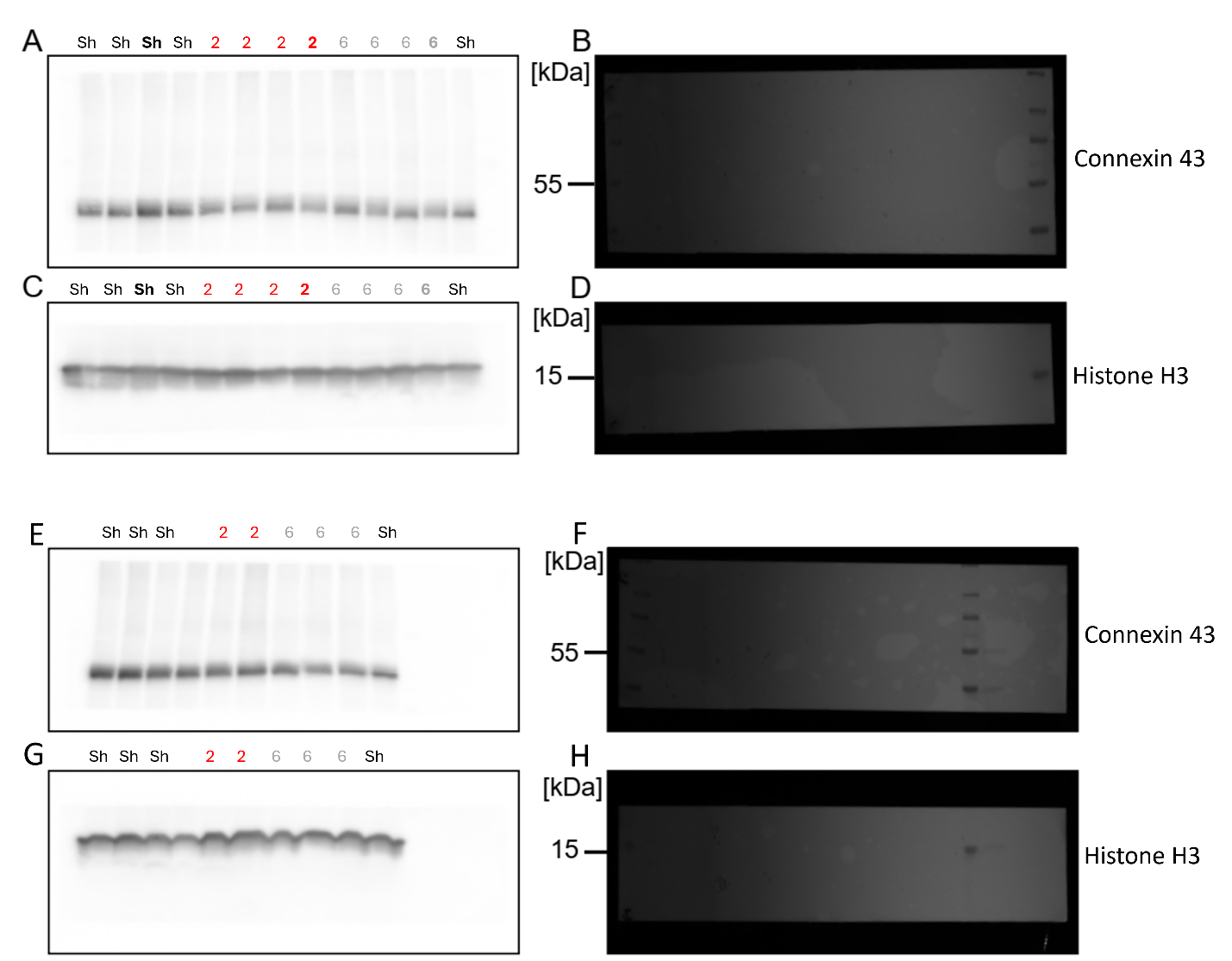


**Figure S6. Western blot analysis of the Cx43 in AAC animals**

Protein expression of Cx43 in left ventricle normalized to histone H3. Uncropped Western blot images of Cx43 detected using anti-Cx43 antibody Sigma #C6219, dilution 1:8000. Membranes were cut in half for parallel processing, and each half was incubated with a primary antibody for either Cx43^S368^ (A,B, E,F) or histone H3 (Abcam #ab1791, dilution 1:2500; C,D,G,H). Chemiluminiscence signal (A, C, E, and G) and corresponding visible light scans (B, D, F, and H) are shown for both membranes. The Sham samples at the end of the membrane were loaded onto both membranes to serve as an internal reference for comparison across blots. One sample from the AAC-PD2 group, which lacks labeling in the figure, was identified as an outlier and excluded from further analysis.

Molecular weight markers are visible in panel B, D, F, and H, with positions indicated on the membrane.

Sample groups are identified by the following colors: Sham - Sh, in black, AAC-PD2 – 2, in red, and AAC-PD6 - 6, in grey. Representative samples included in Figure 8 are shown in bold.

Figure S7


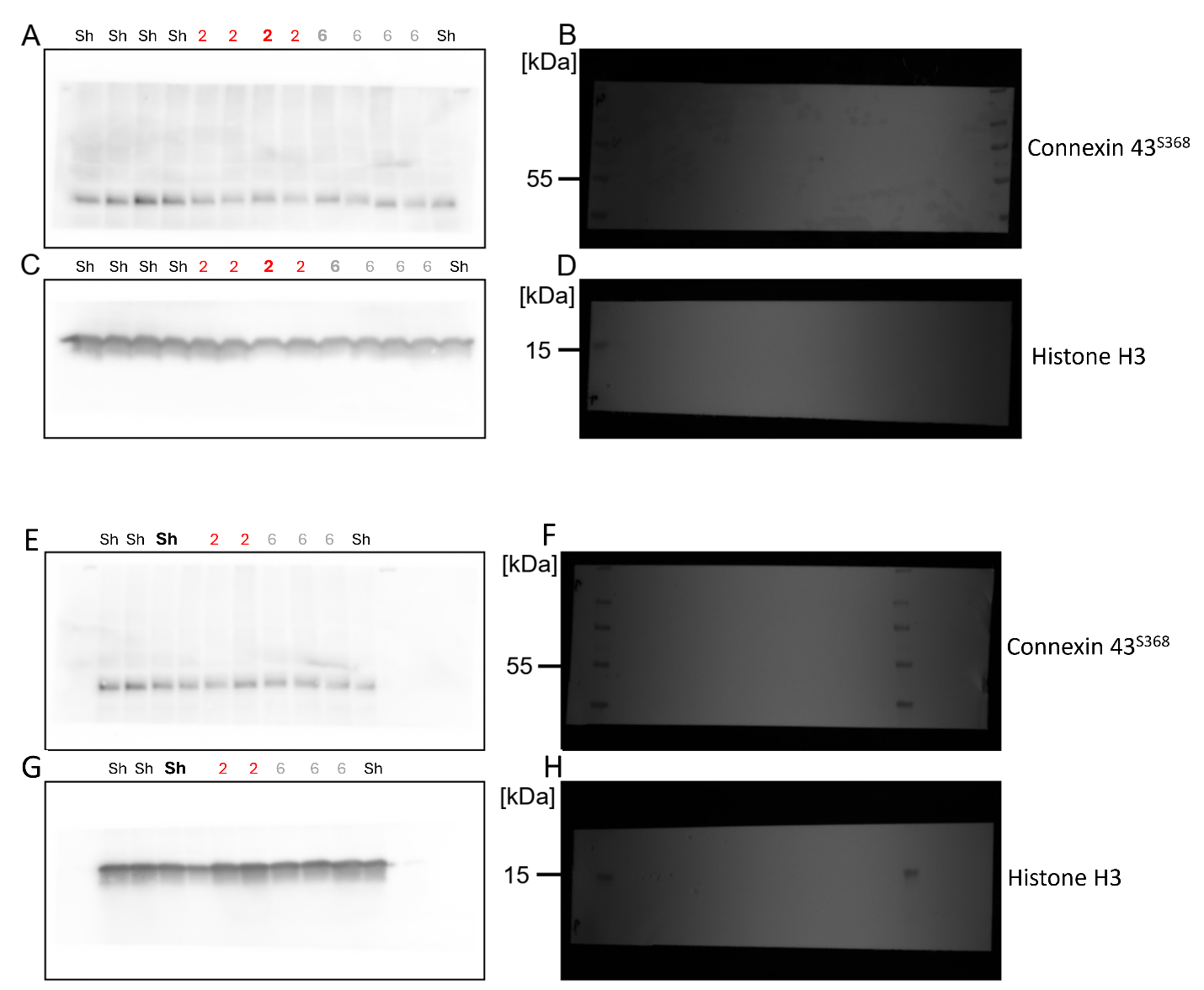


**Figure S7. Western blot analysis of the Cx43^S368^ in AAC animals**

Protein expression of Cx43 **^S368^** in left ventricle was normalized to histone H3. Uncropped Western blot images of phosphorylated Cx43 **^S368^** detected using anti-Cx43 antibody Cell Signaling Technology #3511, dilution 1:5000. Membranes were cut in half for parallel processing, and each half was incubated with a primary antibody for either Cx43^S368^ (A, B, E and F) or histone H3 (Abcam #ab1791, dilution 1:2500 C,D,G,H). Chemiluminiscence signal (A, C, E, and G) and corresponding visible light scans (B, D, F, and H) are shown for both membranes. The Sham samples at the end of the membrane were loaded onto both membranes to serve as an internal reference for comparison across blots. One sample from the AAC-PD2 group, which lacks labeling in the figure, was identified as an outlier and excluded from further analysis.

Molecular weight markers are visible in panel B, D, F, and H, with positions indicated on the membrane.

Sample groups are identified by the following colors: Sham - Sh, in black, AAC-PD2 – 2, in red, and AAC-PD6 - 6, in grey. Representative samples included in Figure 8 are shown in bold.

Figure S8


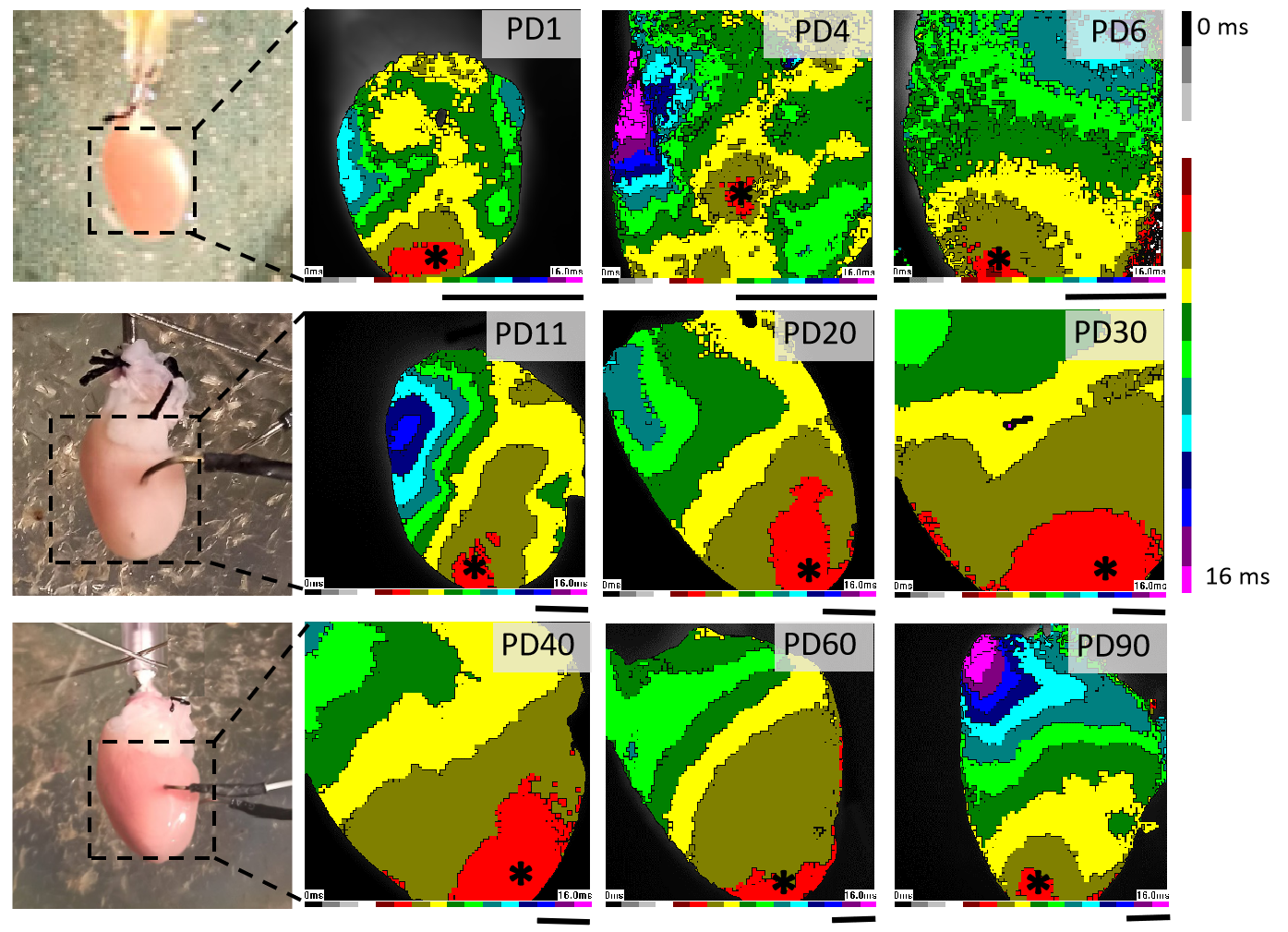


F**igure S6. Spontaneous activation pattern in the PD1-PD90**

Visualized LV pattern after spontaneous activation (first column shows area of the analyzed LV). An asterisk indicates the earliest site of activation. Color bands represent 1 ms.

**Supplementary Tables**

**Table S1. Similar cardiomyocyte size in AAC-PD2 and Sham rats**

|  | Sham | AAC-PD2 | AAC-PD6 |
| --- | --- | --- | --- |
| Cardiomyocyte width, μm | 8.3 ± 0.71 | 7.89 ± 0.71 | 9.77 ± 0.86** |
| Cardiomyocyte volume, μm^3^ | 3366 ± 461 | 3430 ± 629 | 5375 ± 13949*** |

Data are expressed as mean ± SD; one-way ANOVA with Tukey multiple comparisons test, ***p < 0.05, **p < 0.01 vs. Sham.

Table S2. Additional echocardiographic data from the AAC animals

|  | Sham | AAC-PD2 | AAC-PD6 |
| --- | --- | --- | --- |
| LV Geometry |  |  |  |
| AWTs, mm | 1.79 ± 0.12 | 2.05 ± 0.23* | 2.03 ± 0.27 |
| PWTs, mm | 1.69 ± 0.12 | 1.97 ± 0.22** | 1.91 ± 0.18* |
| FWs, mm | 1.38 ± 0.12 | 1.86 ± 0.40** | 1.82 ± 0.36* |
| IVSs, mm | 1.22 ± 0.07 | 1.45 ± 0.19* | 1.38 ± 0.25 |
| LVDs, mm | 2.85 ± 0.39 | 4.09 ± 1.12* | 3.99 ± 0.93* |
| Pulmonary artery flow |  |  |  |
| V_max_, m/s | 0.73 ± 0.08 | 0.74 ± 0.11 | 0.69 ± 0.10 |
| V_mean_, m/s | 0.30 ± 0.05 | 0.28 ± 0.06 | 0.25 ± 0.04 |
| AT_p_, ms | 25.3 ± 2.6 | 28.4 ± 4.0 | 28.1 ± 2.9 |
| ET_p_, ms | 88.2 ± 4.1 | 82.8 ± 7.4 | 81.8 ± 9.1 |
| Mitral flow |  |  |  |
| FT, ms | 52.9 ± 3.3 | 52.9 ± 10.6 | 47.9 ± 8.9 |
| IVCT, ms | 16.1 ± 4.6 | 19.3 ± 5.4 | 15.3 ± 3.8 |
| ET_m_, ms | 65.8 ± 7.3 | 60.8 ± 11.7 | 58.7 ± 14.7 |

AWTs, systolic anterior wall thickness; PWTs, systolic posterior wall thickness, FWs, systolic free wall thickness; IVSs, systolic interventricular septum thickness; LVDs, systolic left ventricle diameter; V_max_, maximal pulmonary artery blood flow; V_mean_, mean pulmonary artery blood flow; AT_p_, acceleration time in pulmonary artery; ET_p_, ejection time in pulmonary artery; FT, left ventricle filling time; IVCT, isovolumic contraction time, ET_m_, ejection time to aorta. Data are expressed as mean ± SD; one-way ANOVA with Tukey multiple comparisons test, *p < 0.05, **p < 0.01 vs. Sham animals.

**Supplementary Methods**

Phenotype progression analyses by echocardiography

In the pulmonary artery, Doppler pulse wave measurement was used to assess acceleration time (ATp), ejection time (ETp) and maximal and mean velocity of blood flow (Vp_max_ and Vp_mean_, respectively). At the level of the mitral annulus, assessements were made for filling time (FT), isovolumic contraction time (IVCT), ejection time (ETm) and IVRT.

Based on LV geometry, the following parameters were calculated:

Relative wall thickness (RWT) = 100*(AWTd+PWTd)/LVDd

Fractional shortening (FS) = 100*(LVDd-LVDs)/LVDd

Stroke volume (SV) = (π/3)*(LVDd^3^-LVDs^3^)

Ejection fraction (EF) = (π/3)*(LVDd^3^-LVDs^3^)/LVDd^3^

Cardiac output (CO) = SV*HR

Electrocardiographic analysis

The ECG records were analyzed using LabChart Pro software (ADInstruments, USA). The following parameters were calculated:

P wave – onset and end defined as the intersection with the isoelectric line

PR interval – from the start of the P wave to the R peak of the QRS complex

QRS complex – from the first deflection of the QRS to the J wave or the intersection with the isoelectric line at the end of the QRS complex

QT – from the first deflection of the QRS to the intersection with the isoelectric line at the end of the T wave

QTc – QT interval corrected for heart rate using Bazett’s formula

Western blot analyses

The amounts of the Cx43 and Cx43^S368^ in AAC animals were detected by western blotting. Samples were homogenized in ice-cold RIPA lysis buffer supplemented with PhosSTOP™ phosphatase inhibitor cocktail (Roche, Switzerland) and cOmplete™ protease inhibitor cocktail (Roche, Switzerland), using 3 mm tungsten carbide beads (Qiagen, Germany) in a TissueLyser II system (Qiagen, Germany). Homogenates were incubated for 30 minutes at 4 °C with gentle rotation. Beads were then removed, and lysates were centrifuged at 12,000 × g for 20 minutes at 4 °C. Supernatants were collected, and total protein concentrations were determined using the Pierce™ BCA Protein Assay Kit (Thermo Fisher Scientific, USA) according to the manufacturer’s instructions. For each sample, 20 µg of total protein was separated on 4–20% Mini-PROTEAN® TGX™ precast SDS-PAGE gels (Bio-Rad, USA) using Tris-Glycine running buffer (25 mM Tris, 192 mM glycine, 0.1% SDS, pH 8.3). Proteins were transferred to 0.2 µm nitrocellulose membranes using the Trans-Blot® Turbo™ Transfer System and corresponding transfer packs (Bio-Rad, USA). Membranes were stained with Ponceau S solution (0.1% Ponceau S in 5% acetic acid) for 4 minutes to verify uniform protein transfer, imaged and subsequently rinsed with distilled water. Membranes were cut to allow for separate detection of the protein of interest and the loading control. Blocking was performed for 1 hour at room temperature in 5% bovine serum albumin (BSA; Sigma-Aldrich, USA) in TBS-T (20 mM Tris-HCl, 150 mM NaCl, 0.1% Tween 20, pH 7.6). Membranes were incubated overnight at 4 °C with the following primary antibodies diluted in 5% BSA in TBS-T: rabbit anti-Cx43 (1:8000, Sigma-Aldrich #C6219, USA), rabbit anti-phospho-Cx43 (1:5000, Cell Signaling Technology #3511, USA), and rabbit anti-histone H3 (1:2500, Abcam #ab1791, UK). After three 15-minute washes in TBS-T, membranes were incubated with HRP-conjugated goat anti-rabbit secondary antibody (1:10000, Abcam #ab6721, UK) for 1 hour at room temperature. Subsequently, membranes were washed three times for 15 minutes each in fresh TBS-T. Chemiluminescent detection was performed using SuperSignal™ West Pico PLUS Chemiluminescent Substrate (Thermo Fisher Scientific, USA), and membranes were imaged with an Azure C600 imaging system (Azure Biosystems, USA). Target protein signals were normalized to their loading controls/total protein and reference sample.
